# Etoricoxib–Betamethasone Combination Attenuates Inflammatory Nociception and Edema in Adjuvant-Induced Arthritis via Cytokine and Macrophage Axis Modulation

**DOI:** 10.3390/ph19081235

**Published:** 2026-08-06

**Authors:** José Pérez-Urizar, Irma Torres-Roque, Velia Verónica Rangel-Ramírez, Juan Pablo Castillo-Enriquez, Héctor Lee-Rangel, Kevin F. Rios-Brito, Darío A. Morales-Martínez, Jorge González-Canudas

**Affiliations:** 1 Facultad de Ciencias Químicas, Universidad Autónoma de San Luis Potosí, Av. Dr. Manuel Nava #6, Zona Universitaria, San Luis Potosí 78210, Mexico; 2 FS Scientia Pharma S.A. de C.V., Fray Diego de la Magdalena 630, Colonia Jardín, San Luis Potosí 78270, Mexico; irma.torres@fsscientiapharma.com; 3 Centro Regional de Biociencias, Universidad Autónoma de San Luis Potosí, San Luis Potosí 78321, Mexico; veronica.rangel@uaslp.mx (V.V.R.-R.); jpce213@gmail.com (J.P.C.-E.); hector.lee@uaslp.mx (H.L.-R.); 4 Dirección de Investigación y Desarrollo, Laboratorios Silanes S.A. de C.V., Ciudad de México 11000, Mexico; krios@silanes.com.mx (K.F.R.-B.); dmoralesm@silanes.com.mx (D.A.M.-M.); jogonzalez@silanes.com.mx (J.G.-C.); 5 Instituto Mexicano del Seguro Social, Centro Médico Nacional Siglo XXI, Ciudad de México 06720, Mexico

**Keywords:** adjuvant-induced arthritis, etoricoxib, betamethasone, inflammatory nociception, cyclooxygenase-2, cytokines

## Abstract

**Background/Objectives:** Acute inflammatory episodes demand rapid symptom control while limiting systemic exposure. We assessed whether co-therapy with the selective cyclooxygenase-2 (COX-2) inhibitor etoricoxib and the corticosteroid betamethasone provides antinociceptive and anti-edema activity in a rat model with complete Freund’s adjuvant-induced arthritis (AIA). **Methods:** Male Wistar rats (*n* = 10/group) were allocated to seven groups: Intact, AIA disease control, indomethacin 5 mg/kg, etoricoxib 8 mg/kg, betamethasone 0.022 mg/kg, low-dose combination (4 + 0.011 mg/kg), and full-dose combination (8 + 0.022 mg/kg), administered orally once daily from Days 4 to 28. Paw edema, von Frey withdrawal thresholds, and clinical arthritis score were assessed longitudinally as area under the curve (AUC) values. Terminal joint tissues were profiled for cytokines, prostaglandin pathway mediators, and immune cell markers. **Results:** Both combinations reduced edema and improved mechanical thresholds. The full-dose combination exceeded either monotherapy regarding mechanical sensitivity and the arthritis index, consistent with additive activity. The low-dose combination matched full-dose monotherapies, consistent with a dose-reduction effect. Biomarker shifts indicated attenuated prostaglandin signaling and a pro-resolving cytokine balance. **Conclusions:** These findings support the further evaluation of etoricoxib–betamethasone co-therapy for acute inflammatory conditions.

## 1. Introduction

Acute inflammatory pain flares—such as gout attacks, acute deteriorations of rheumatoid arthritis, and sudden exacerbations of osteoarticular or musculoskeletal disorders—require fast symptom control and must often be managed in patients with cardiometabolic comorbidities and polypharmacy. Rheumatoid arthritis (RA) affects an estimated 0.5% of adults worldwide on a pooled period prevalence basis, with substantial regional variation and a female-to-male ratio of approximately 3:1 [1]. Gout—the most prevalent form of inflammatory arthritis worldwide—affects 1–4% of adults in high-income countries, with its incidence and prevalence rising steeply with age [2]. In the pathogenesis of gout, the prototypical acute inflammatory flare involves hyperuricemia and the deposition of monosodium urate crystals in articular structures, culminating in an innate immune response mediated by the NLRP3 inflammasome, with interleukin (IL)-1β acting as a central effector cytokine [3,4]. Current guideline-supported options for acute gout include non-steroidal anti-inflammatory drugs (NSAIDs), colchicine, and systemic or intra-articular corticosteroids, which may be used as monotherapy or in combination depending on the disease severity, risk–benefit considerations, and contraindications [5,6,7].

The immunopathology of RA involves an initiating phase of T-cell- and B-cell-mediated autoimmune activation, followed by sustained synovial inflammation driven by pro-inflammatory cytokines—principally TNF-α, IL-1β, and IL-6—acting through nuclear factor-kappa B (NF-κB) and activator protein-1 (AP-1) transcriptional programs in fibroblast-like synoviocytes and infiltrating macrophages [8]. A key amplification loop involves the interleukin-23/interleukin-17 (IL-23/IL-17) axis, in which IL-23 promotes Th17 cell proliferation and IL-17 production, which, in turn, upregulates TNF-α, IL-1β, and receptor activator of nuclear factor kappa-B ligand (RANKL) in synovial fibroblasts, exacerbating synovial inflammation and joint destruction through NF-κB-dependent signaling [9,10]. This cytokine network and its downstream engagement of osteoclastogenic and prostaglandin-synthesizing pathways provides the mechanistic rationale for the terminal mediator panel assessed in the present study.

Updated rheumatoid arthritis guidelines allow for the concomitant use of NSAIDs and systemic corticosteroids as an adjunctive symptomatic therapy, particularly as a short-term bridge treatment when initiating or adjusting disease-modifying antirheumatic drugs (DMARDs), with the aim of controlling pain and inflammation while waiting for the DMARD to take full effect. However, in light of safety considerations, this strategy is not recommended for long-term management and should be limited to the lowest effective doses and the shortest feasible duration [11,12,13].

The specific rationale for pairing an NSAID with a corticosteroid, rather than relying on colchicine or either drug class alone, follows from their distinct and complementary mechanisms of action. Colchicine inhibits microtubule polymerization, thereby blocking neutrophil chemotaxis, NLRP3 inflammasome assembly, and IL-1β release, and is recommended as a first-line option specifically for gout flares presenting within 36 h of onset in patients without contraindications (notably, severe renal or hepatic impairment, given its narrow therapeutic index and risk of accumulation) [5]. Corticosteroids act through cytosolic glucocorticoid receptors that, upon ligand binding and nuclear translocation, transrepress NF-κB and AP-1 transcriptional programs, broadly suppressing cytokine transcription and inducing anti-inflammatory mediators such as IL-10 and annexin-1 [14,15]. NSAIDs and corticosteroids are therefore both first-line, guideline-endorsed options with a broader eligible population than colchicine, and their distinct molecular targets (direct enzymatic prostaglandin blockade versus broad transcriptional cytokine suppression) support pairing rather than substitution. Clinically, an insufficient response to monotherapy is recognized when pain, swelling, or functional impairment persists beyond 24–48 h despite an adequate dose of a single first-line agent, when dose escalation is precluded by contraindications or adverse event burden, or when the flare recurs during initial treatment [5,7].

The most recent guidance on acute gout flares from the National Institute for Health and Care Excellence (NICE NG219, 2022) reinforces the central roles of NSAIDs, colchicine, and short-course oral corticosteroids as first-line options, with explicit consideration of combination strategies when initial monotherapy is insufficient or contraindicated [16]. This guideline-level endorsement of combination practice is consistent with patterns observed in large pharmacy claim datasets, where many patients with acute flares receive overlapping prescriptions of NSAIDs and oral corticosteroids—particularly when cardiometabolic comorbidities, chronic kidney disease, or peptic ulcer disease limit the use of either drug class at full monotherapy doses. Clinical and regulatory momentum has therefore shifted toward dose-rationalized combination therapy as a means of preserving efficacy while reducing adverse event exposure; however, this clinical practice has not been accompanied by systematic preclinical pharmacological characterization of specific NSAID–corticosteroid pairings.

A recent systematic review and network meta-analysis of randomized controlled trials evaluating NSAIDs and corticosteroids as symptomatic therapy in rheumatoid arthritis identified only 26 eligible trials over more than two decades, with meta-analyses being feasible only for NSAIDs. The evidence base for prednisolone or prednisone proved insufficient to support quantitative network meta-analytic pooling—that is, too few directly comparable randomized controlled trials with compatible designs and endpoints were available to combine statistically—and no trial directly compared a fixed NSAID–corticosteroid combination against either monotherapy [17]. This evidence gap is mirrored at the preclinical level; in particular, while individual NSAIDs and corticosteroids have been extensively characterized in animal models of inflammatory arthritis, head-to-head preclinical comparisons of specific COX-2 inhibitor + corticosteroid pairings, including parallel quantification of behavioral nociception, edema, terminal cytokine profiles, and prostaglandin pathway mediators, remain scarce. The present work addresses this preclinical evidence gap for the specific pairing of etoricoxib and betamethasone, both of which have established clinical use as monotherapies but whose combined pharmacodynamic profile has not previously been systematically characterized in a standardized inflammatory arthritis model.

NSAIDs commonly recommended for acute gout and rheumatoid arthritis include naproxen, indomethacin, ibuprofen, diclofenac, and the selective COX-2 inhibitors celecoxib and etoricoxib; it should be noted that selective COX-2 inhibitors are increasingly favored in patients with elevated upper gastrointestinal risk, given their improved tolerability relative to nonselective agents [18]. Etoricoxib has demonstrated clinical efficacy comparable to indomethacin in the treatment of acute gouty arthritis [19]. Likewise, in patients with rheumatoid arthritis, once-daily etoricoxib has shown efficacy comparable to twice-daily naproxen [20]. Etoricoxib has a favorable gastrointestinal safety profile, with comparative studies reporting a lower incidence of gastrointestinal adverse events and fewer discontinuations due to dyspepsia than with nonselective NSAIDs; however, as with other COX-2 inhibitors, cardiovascular safety considerations warrant clinical monitoring [18]. Nonselective NSAIDs such as indomethacin, by contrast, carry a comparatively higher burden of gastropathy, ulceration, and renal effects.

Betamethasone is a synthetic corticosteroid and stereoisomer of dexamethasone that exerts potent glucocorticoid activity with broad anti-inflammatory and immunosuppressive effects and minimal mineralocorticoid activity [14,15]. Like other synthetic corticosteroids, it suppresses the formation, release, and activity of endogenous inflammatory mediators, including prostaglandins, kinins, histamine, lysosomal enzymes, and complement. Corticosteroids also broadly suppress the transcription of pro-inflammatory cytokines including TNF-α, IL-1β, IL-6, and type I interferons, primarily through transrepression of NF-κB and AP-1 transcription factors—a mechanism that complements prostaglandin synthesis inhibition mediated via annexin-1/phospholipase A2 suppression [14,15]. At the molecular level, betamethasone binds intracellular glucocorticoid receptors, modulates gene transcription, inhibits leukotriene and prostaglandin synthesis, and limits inflammatory cell recruitment [14,15]. Oral corticosteroids have shown non-inferior symptom relief versus NSAIDs in pragmatic and equivalence trials [21,22]. However, escalating monotherapy doses to achieve rapid control may increase the burden of adverse events such as hypothalamic–pituitary–adrenal (HPA) axis suppression, hyperglycemia, hypertension, osteoporosis, and infection risk with prolonged exposure, motivating strategies that achieve comparable relief with lower exposure to either drug class.

Here, we tested an oral combination of etoricoxib and betamethasone—both with established clinical use as monotherapies and currently under joint clinical evaluation as a fixed-dose combination in acute inflammatory conditions (NCT06863701) and in a parallel Phase III trial in acute bursitis, tendinitis, and synovitis (NCT07328022)—to determine whether this clinically translatable pairing produces coordinated pharmacodynamic activity at the behavioral and biochemical levels, and to test whether the combination reveals a consistent dose reduction pattern in vivo. Among the many possible NSAID–corticosteroid pairings, this specific combination was selected because it corresponds directly to an active clinical development program, providing a defined translational anchor rather than an arbitrary combinatorial choice. We used a rat model with complete Freund’s adjuvant-induced arthritis (AIA), which has been extensively validated for the assessment of inflammatory joint pain and is recognized as a translationally relevant platform for evaluating anti-arthritic pharmacotherapies, including NSAIDs and corticosteroids [23]. Recent dose characterization work has confirmed that intradermal CFA at 10 mg/mL reliably produces robust mechanical hypersensitivity, sustained paw edema, and histological joint changes that recapitulate key features of rheumatoid arthritis nociception over a 20–28-day observation window [24]. Within this model, we profiled prostaglandin-linked mediators and cytokine-/macrophage-associated readouts as mechanistic correlates [25,26]. We hypothesized that co-therapy would outperform either monotherapy for integrated nociceptive and disease severity endpoints, consistent with their complementary COX-2 and corticosteroid pathways.

## 2. Results

### 2.1. Arthritis Induction with CFA Produced a Robust and Sustained Inflammatory Phenotype

The baseline paw volume at Day 0 was comparable across all seven groups (group means ranged from 1.22 to 1.31 mL), and baseline mechanical withdrawal thresholds and clinical arthritis scores were equivalent, confirming successful pre-treatment randomization. Following intradermal CFA administration on Day 0 with a tail-base booster on Day 4, the AIA-CFA disease control group developed a robust and sustained increase in ipsilateral paw volume (e.g., Day 5: 1.87 ± 0.15 mL; Day 28: 1.89 ± 0.17 mL; mean ± SEM) relative to intact controls (Day 5: 1.10 ± 0.08 mL). In parallel, AIA-CFA animals developed marked mechanical hypersensitivity and elevated clinical arthritis scores that persisted throughout the 28-day observation period. This consistent multimodal phenotype—edema, mechanical hypersensitivity, and clinical arthritis severity—provided the disease severity baseline against which the seven experimental groups were compared across three primary efficacy endpoints (paw edema, mechanical threshold, and arthritis index), each summarized below as a longitudinal time course followed by its integrated area under the curve (AUC) metric. Integrated AUC values for all three endpoints, together with fold change comparisons, are consolidated in Table 1.

**Figure 1:**
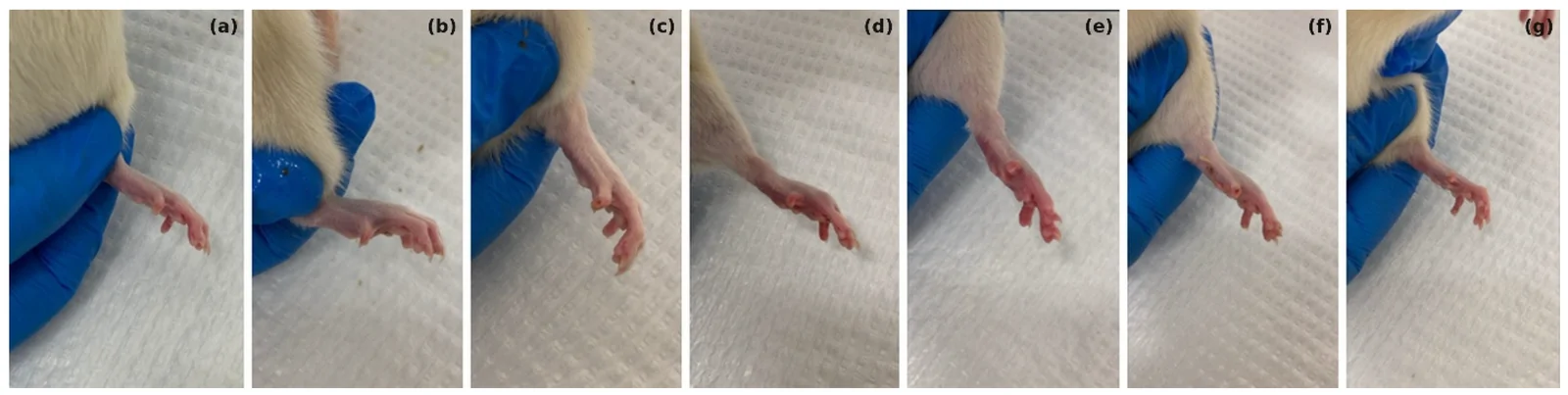
Representative photographs of the ipsilateral hind paw at study completion (Day 28) for each experimental group: (**a**) Intact control; (**b**) AIA-CFA disease control; (**c**) indomethacin (5 mg/kg); (**d**) etoricoxib (8 mg/kg); (**e**) betamethasone (0.022 mg/kg); (**f**) Combo-L (etoricoxib 4 mg/kg + betamethasone 0.011 mg/kg); (**g**) Combo-H (etoricoxib 8 mg/kg + betamethasone 0.022 mg/kg). One representative animal per group is shown.

Representative photographs of the ipsilateral hind paw were obtained for each experimental group at study completion (Day 28), providing direct visual confirmation of the AIA-CFA phenotype and its modulation by treatment (Figure 1).

### 2.2. Etoricoxib–Betamethasone Combinations Reduce Inflammatory Paw Edema

**Figure 2:**
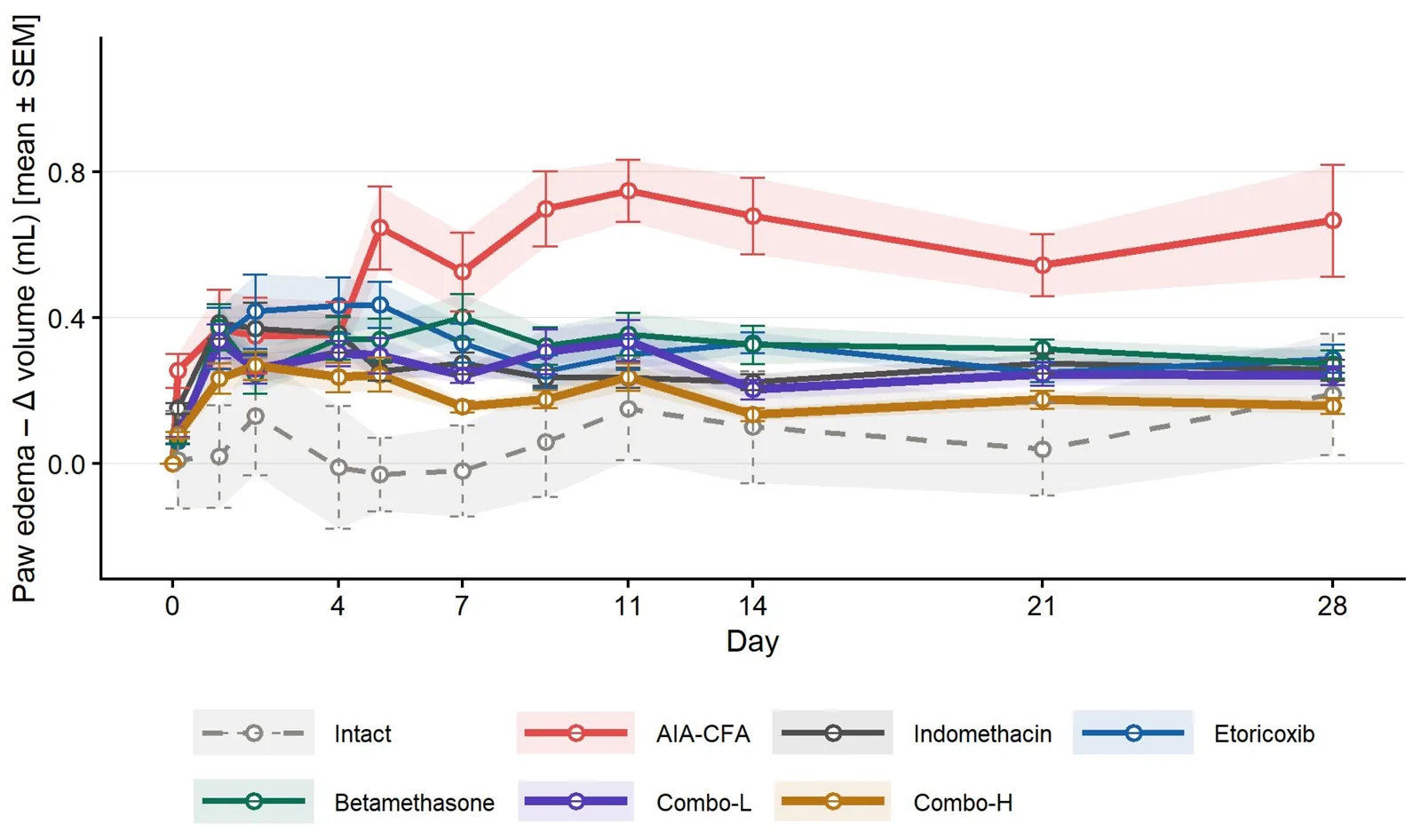
Paw edema time course in the CFA-injected hind paw assessed by water displacement plethysmometry. Data are mean ± SEM; n = 10/group. Groups: Intact control, AIA-CFA disease control, indomethacin (5 mg/kg), etoricoxib (8 mg/kg), betamethasone (0.022 mg/kg), Combo-L (etoricoxib 4 mg/kg + betamethasone 0.011 mg/kg), Combo-H (etoricoxib 8 mg/kg + betamethasone 0.022 mg/kg). Treatments were administered orally once daily from Days 4 to 28.

The longitudinal Δ paw volume profile (Figure 2) shows that, from the first post-treatment assessment on Day 5, all active treatment groups presented visibly lower trajectories than the AIA-CFA disease control. Throughout the 28-day observation period, AIA-CFA animals maintained Δ paw volume values of approximately 0.55–0.75 mL, whereas all animals under active treatments—indomethacin, etoricoxib, betamethasone, Combo-L, and Combo-H—converged at approximately 0.15–0.30 mL by Day 14, with Combo-H consistently maintaining the lowest profile. Paw edema in this model is an established pharmacologically responsive efficacy endpoint, with sensitivity to anti-inflammatory intervention consistently documented across the preclinical literature [23,24].

**Figure 3:**
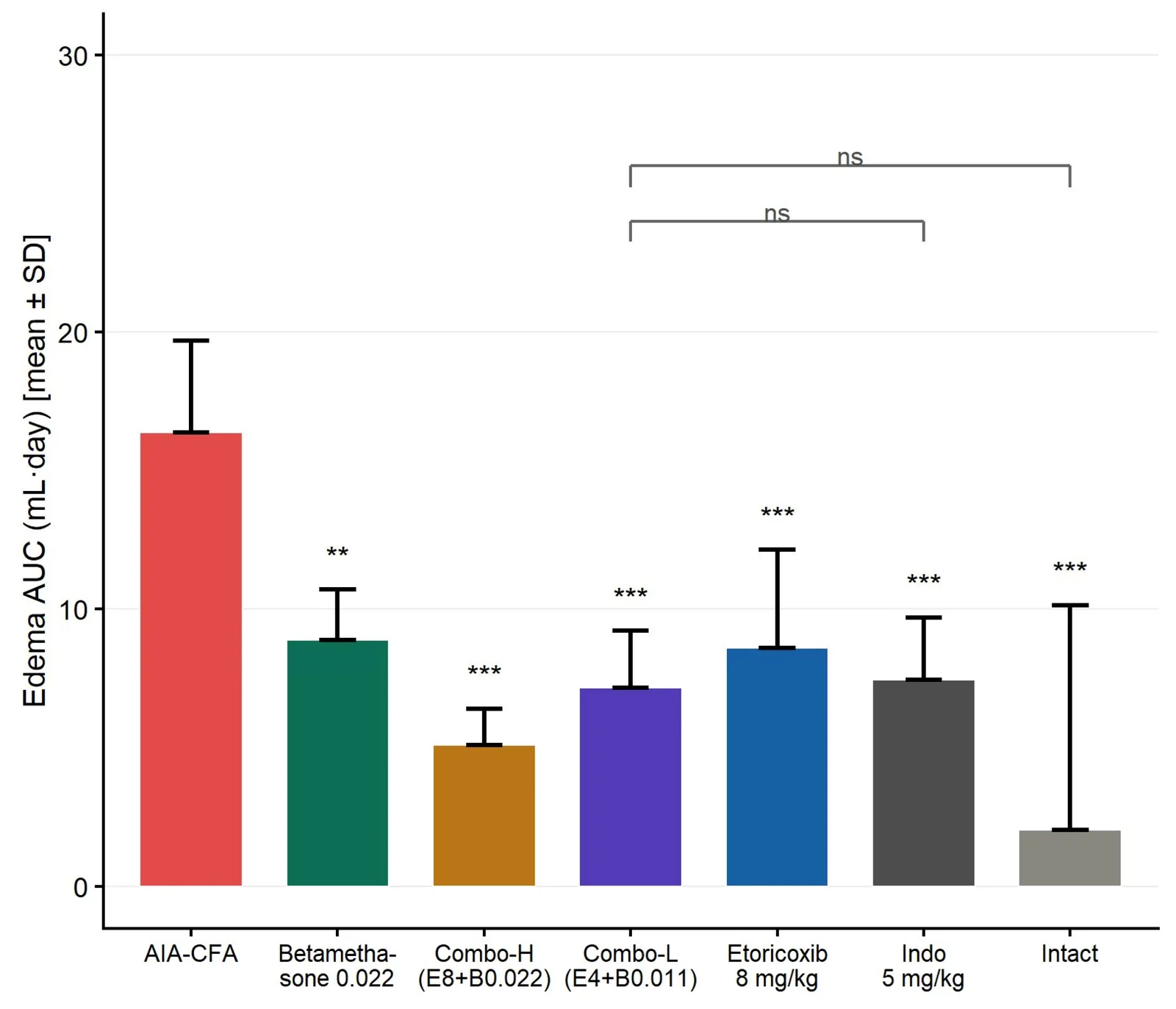
Integrated paw edema burden quantified as area under the curve (AUC) of Δ paw volume versus Day 0. Bars represent mean ± SD; n = 10/group. Significance vs. AIA-CFA disease control (one-way ANOVA, Tukey’s post hoc test): *** *p* < 0.001, ** *p* < 0.01. Tukey-adjusted pairwise comparisons between combination groups and monotherapies were non-significant for all edema AUC contrasts (all *p* > 0.30; see Supplementary Table S1). ns = not significant (*p* ≥ 0.05). ‘E’ and ‘B’ in group labels denote etoricoxib and betamethasone dose (mg/kg), respectively; e.g., Combo-H (E8 + B0.022) = etoricoxib 8 mg/kg + betamethasone 0.022 mg/kg.

Quantification of the integrated edema burden as AUC of Δ paw volume versus Day 0 (Figure 3; Table 1) confirmed and refined these observations. Edema AUC was markedly elevated in the AIA-CFA disease control (16.37 ± 3.32 mL·day; mean ± SD; n = 10) and was significantly reduced by indomethacin (7.45 ± 2.26 mL·day; *p* = 0.0001 vs. AIA-CFA), etoricoxib (8.59 ± 3.55 mL·day; *p* = 0.0007), and betamethasone (8.89 ± 1.82 mL·day; *p* = 0.0013). Both combinations achieved numerically greater reductions: Combo-L reached 7.16 ± 2.06 mL·day (*p* < 0.0001 vs. AIA-CFA), while Combo-H achieved the lowest edema AUC of any active group (5.09 ± 1.33 mL·day; *p* < 0.0001 vs. AIA-CFA), representing a 68.9% reduction relative to the disease control. Expressed as fold change versus AIA-CFA (Table 1), edema AUC was reduced to 0.46-fold by indomethacin, 0.52-fold by etoricoxib, 0.54-fold by betamethasone, 0.44-fold by Combo-L, and 0.31-fold by Combo-H; expressed as fold change versus indomethacin, Combo-H reached 0.68-fold (i.e., a further 32% reduction beyond the positive control). However, Tukey-adjusted pairwise comparisons between the combinations and the individual monotherapies did not reach statistical significance (Combo-H vs. etoricoxib *p* = 0.4021; Combo-H vs. betamethasone *p* = 0.3052; Combo-L vs. etoricoxib *p* = 0.9804; Combo-L vs. betamethasone *p* = 0.9509; for the complete pairwise table, see Supplementary Table S1). This pattern indicates that paw edema is the efficacy endpoint with the smallest between-treatment dynamic range in this 28-day model, namely, all active treatments, alone or in combination, achieved near-equivalent suppression.

### 2.3. The Full-Dose Combination Achieves Superior Restoration of Mechanical Withdrawal Threshold

**Figure 4:**
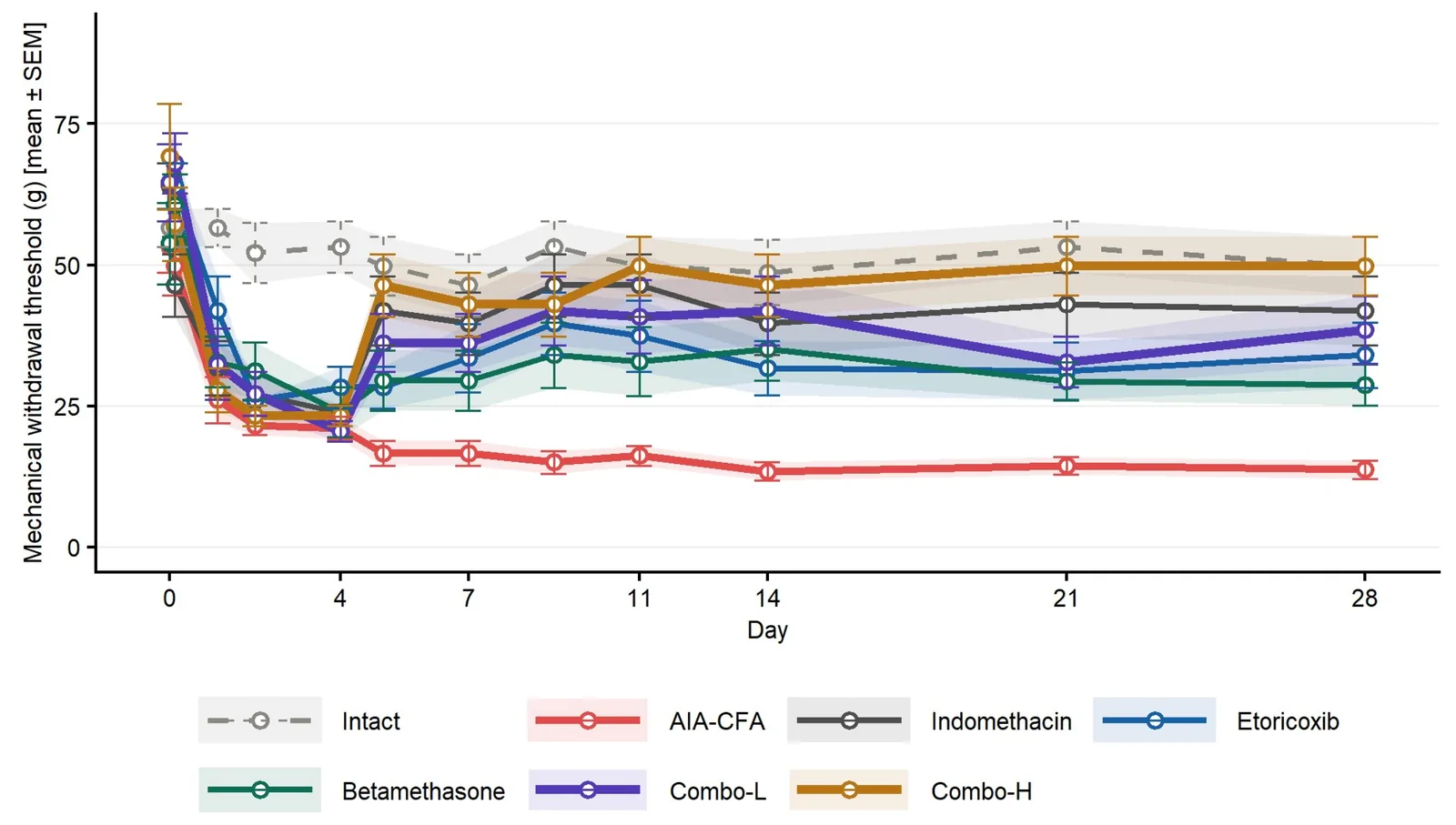
Mechanical withdrawal threshold (von Frey) time course assessed in the ipsilateral hind paw. Data are mean ± SEM; n = 10/group. Higher values indicate reduced mechanical hypersensitivity. Groups as in Figure 2. Treatments administered orally once daily from Days 4 to 28.

Building on the edema findings above, we next examined mechanical withdrawal threshold—an endpoint that, unlike edema, revealed clear between-treatment differentiation. The longitudinal mechanical withdrawal threshold profile (Figure 4) shows a rapid post-treatment divergence from the AIA-CFA trajectory. AIA-CFA disease controls dropped sharply from baseline values of ~50 g to ~14 g by Day 7 and remained at this depressed threshold through to Day 28, indicating persistent mechanical hypersensitivity. In contrast, all active groups exhibited a rapid recovery beginning within 24 h of the first dose: by Day 5, Combo-H and indomethacin restored thresholds to ~47–50 g, approaching the intact control level (~53 g), while etoricoxib, betamethasone, and Combo-L produced intermediate restoration to ~30–43 g. This separation was maintained throughout the study. By Day 28, the rank order of restored thresholds was as follows: Intact (~50 g) ≈ Combo-H (~50 g) > Indomethacin (~42 g) > Combo-L (~38 g) > Etoricoxib (~34 g) > Betamethasone (~29 g) >> AIA-CFA (~13 g). Combo-H was the only active treatment that restored the terminal von Frey threshold to within the intact control range (Figure 4); the integrated AUC was significantly higher than either monotherapy (*p* < 0.05), whereas the numerical difference versus indomethacin was not statistically significant (*p* = 0.9023; Supplementary Table S1).

**Figure 5:**
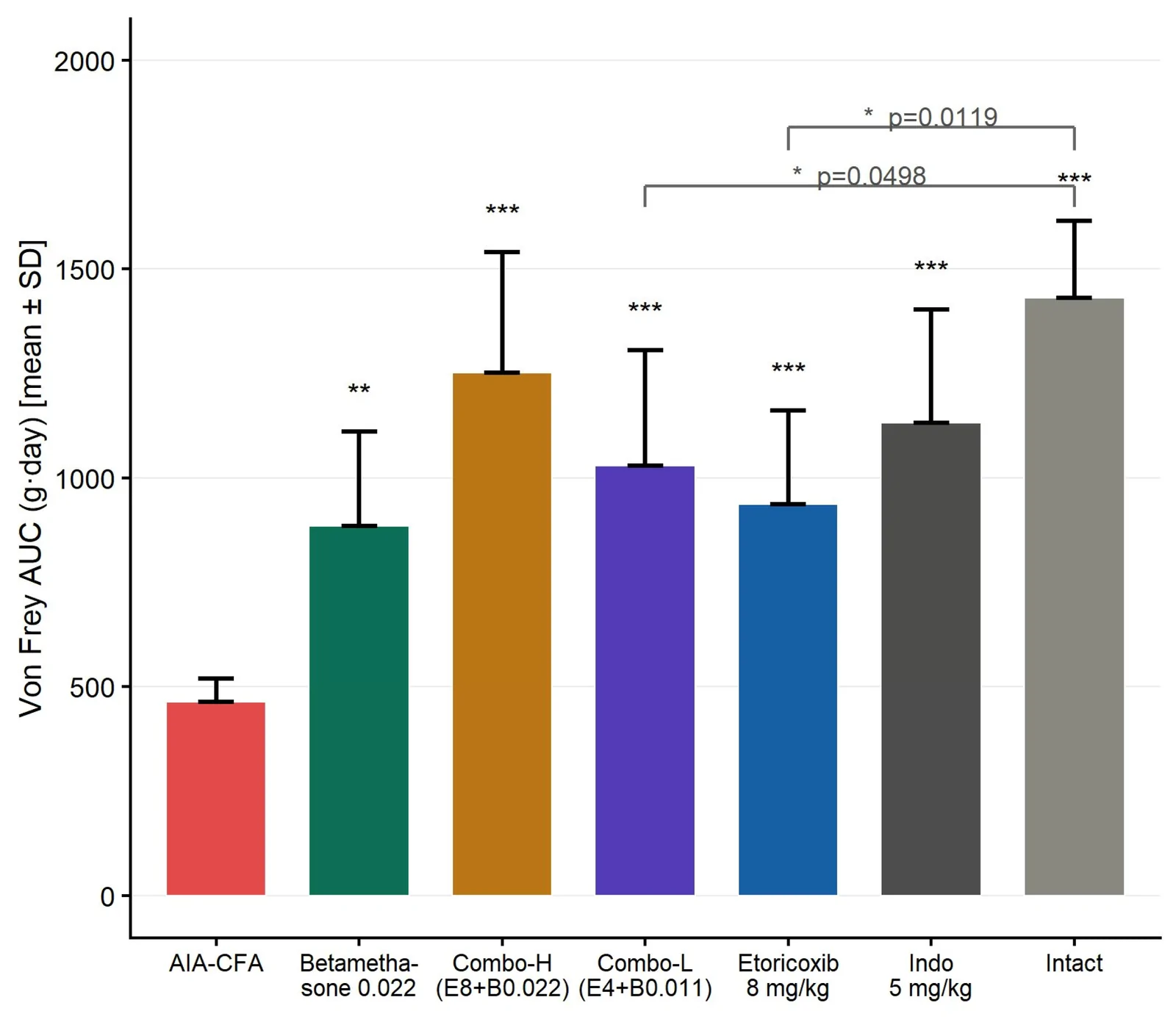
Integrated mechanical sensitivity, quantified as AUC of withdrawal threshold (von Frey). Bars represent mean ± SD; n = 10/group. Significance vs. AIA-CFA disease control (one-way ANOVA, Tukey’s post hoc test): *** *p* < 0.001, ** *p* < 0.01, * *p* < 0.05. Combo-H was significantly greater than etoricoxib (*p* = 0.0498) and betamethasone (*p* = 0.0119); Combo-L did not significantly differ from either monotherapy (all *p* > 0.79). ‘E’ and ‘B’ in group labels denote etoricoxib and betamethasone dose (mg/kg), respectively; e.g., Combo-H (E8 + B0.022) = etoricoxib 8 mg/kg + betamethasone 0.022 mg/kg.

The integrated von Frey AUC analysis (Figure 5; Table 1) provided formal statistical confirmation of this rank order. The threshold AUC was 462.86 ± 56.86 g·day in AIA-CFA versus 1431.73 ± 184.07 g·day in intact controls (*p* < 0.0001), and was significantly increased by all active treatments: indomethacin to 1131.12 ± 273.04 g·day (*p* < 0.0001), etoricoxib to 937.48 ± 223.80 g·day (*p* = 0.0004), and betamethasone to 884.15 ± 226.34 g·day (*p* = 0.0024). Combo-L improved the threshold AUC to 1028.86 ± 276.61 g·day (*p* < 0.0001 vs. AIA-CFA) and was statistically equivalent to both full-dose monotherapies (Combo-L vs. etoricoxib *p* = 0.9735; Combo-L vs. betamethasone *p* = 0.7988). Crucially, Combo-H produced the greatest restoration (1252.01 ± 288.89 g·day; *p* < 0.0001 vs. AIA-CFA; fold change vs. AIA-CFA = 2.70, Table 1) and was significantly greater than etoricoxib (*p* = 0.0498) and betamethasone (*p* = 0.0119). These pairwise contrasts (which are annotated directly in Figure 5) provide formal statistical support for the additive antinociceptive activity of the two mechanistically complementary agents administered at their full doses.

### 2.4. The Full-Dose Combination Produces the Greatest Reduction in Clinical Arthritis Severity

**Figure 6:**
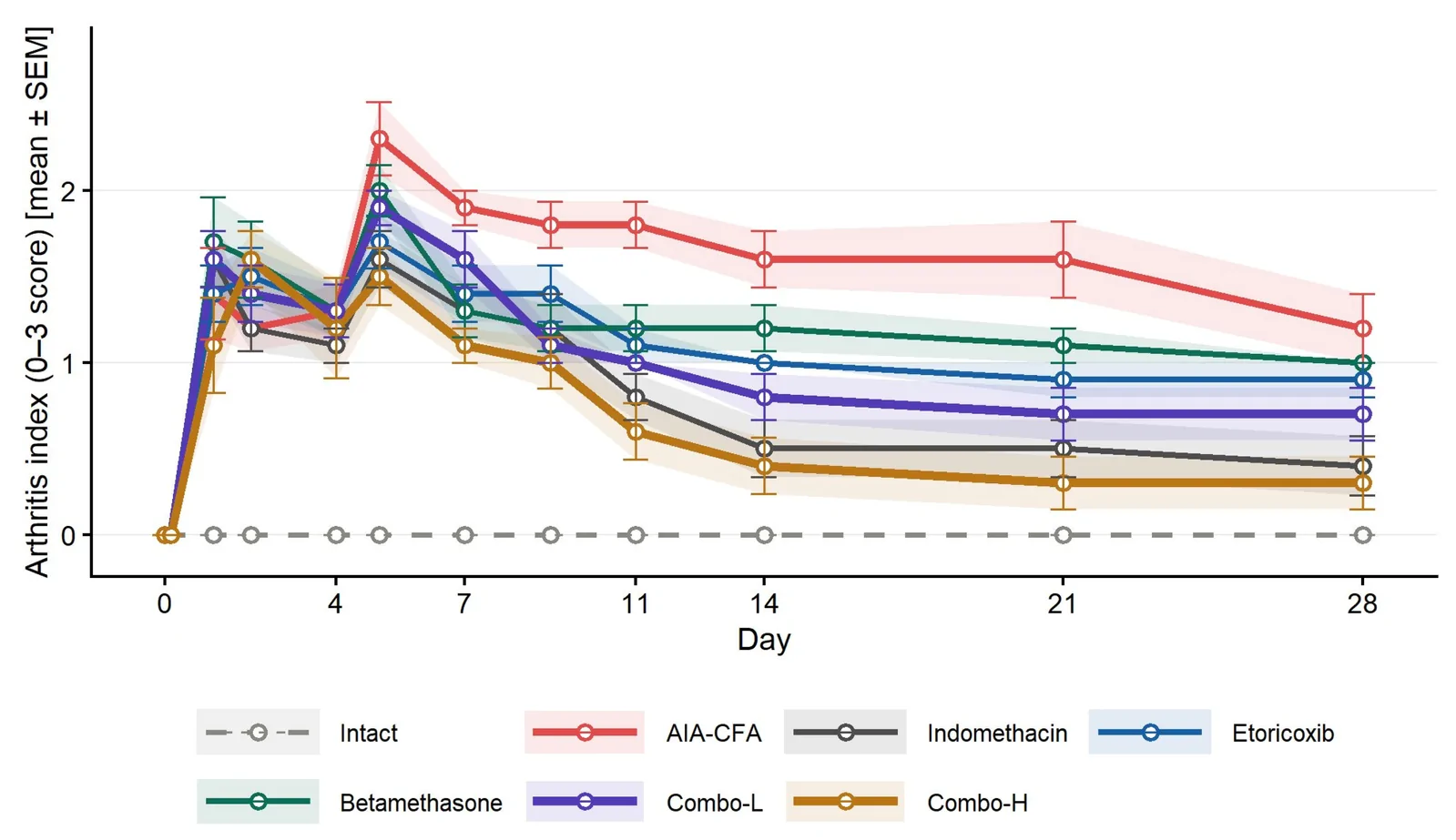
Clinical arthritis index time course. Arthritis severity was scored on an ordinal scale (0 = normal; 1 = mild edema/erythema; 2 = moderate swelling and redness; 3 = severe swelling and deformity). Data are mean ± SEM; n = 10/group. Groups as in Figure 2.

The clinical arthritis index provides an integrated, ordinal measure of joint inflammation which his complementary to the two continuous endpoints discussed above. The longitudinal arthritis index profile (Figure 6) shows a characteristic bimodal response in AIA-CFA disease controls: an initial peak at ~1.7 by Day 1 following the primary CFA injection, a partial decline at Day 4, and a secondary peak at ~2.3 on Day 5 following the booster, with gradual decline to ~1.2 by Day 28. Active treatments produced distinct trajectories. Combo-H showed the most rapid and complete reduction, stabilizing at ~0.3 from Day 14 onward, thus approaching the intact baseline. Indomethacin followed a similar trajectory, ending at ~0.4 by Day 28. Combo-L showed an intermediate response (~0.7 at Day 28), while etoricoxib and betamethasone monotherapies produced more modest declines, ending at ~0.9 and ~1.0, respectively, by Day 28.

**Figure 7:**
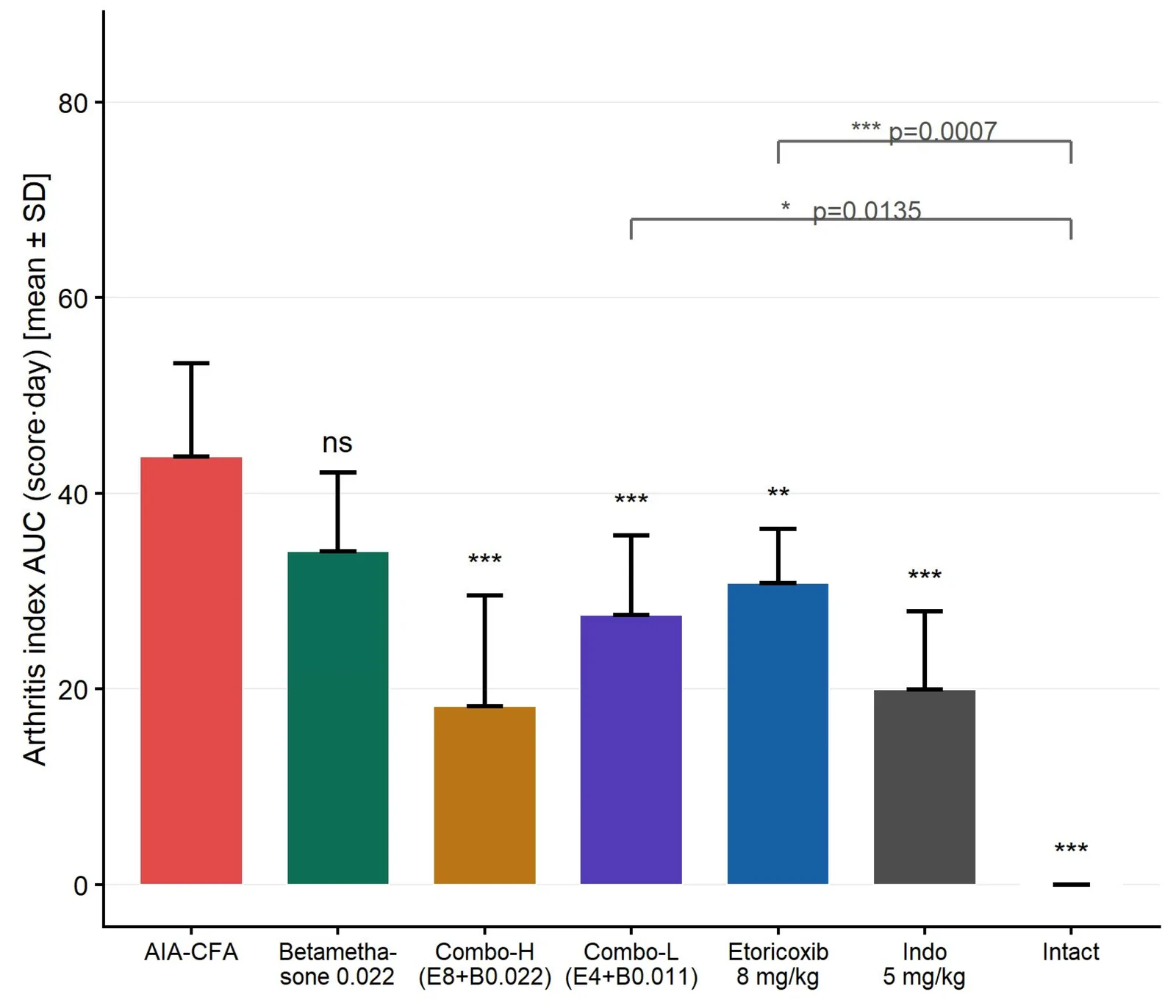
Integrated arthritis severity quantified as AUC of the clinical arthritis index. Bars represent mean ± SD; n = 10/group. Significance vs. AIA-CFA disease control (one-way ANOVA, Tukey’s post hoc test): *** *p* < 0.001, ** *p* < 0.01, * *p* < 0.05, ns = not significant (*p* ≥ 0.05). Combo-H was significantly lower than etoricoxib (*p* = 0.0135) and betamethasone (*p* = 0.0007); Combo-L did not significantly differ from either monotherapy (all *p* > 0.54). ‘E’ and ‘B’ in group labels denote etoricoxib and betamethasone dose (mg/kg), respectively; e.g., Combo-H (E8 + B0.022) = etoricoxib 8 mg/kg + betamethasone 0.022 mg/kg.

AUC analysis of the integrated arthritis index (Figure 7; Table 1) revealed the most pronounced between-treatment differentiation of any efficacy endpoint in this study. The AIA-CFA disease control reached an arthritis AUC of 43.74 ± 9.51 score·day. Indomethacin reduced this to 19.93 ± 8.01 (*p* < 0.0001 vs. AIA-CFA) and etoricoxib produced a significant but smaller reduction (30.77 ± 5.55; *p* = 0.0094), whereas betamethasone monotherapy produced only a modest, non-significant reduction (34.04 ± 8.08; *p* = 0.1097). Combo-L significantly reduced the arthritis AUC to 27.56 ± 8.12 (*p* = 0.0005 vs. AIA-CFA), with non-significant Tukey-adjusted contrasts when compared with monotherapies (vs. etoricoxib *p* = 0.9712; vs. betamethasone *p* = 0.5402). Combo-H achieved the largest reduction observed in the study (18.23 ± 11.29; *p* < 0.0001 vs. AIA-CFA; fold change vs. AIA-CFA = 0.42, Table 1) and was significantly lower than etoricoxib (*p* = 0.0135) and betamethasone (*p* = 0.0007). Notably, Combo-H did not differ significantly from indomethacin (*p* = 0.9991; fold change vs. indomethacin = 0.91; Supplementary Table S1), indicating that the full-dose combination reached the efficacy ceiling defined by the positive control on this endpoint. This pattern reveals the superiority of the full-dose combination over both monotherapies on the arthritis index, paralleling the von Frey AUC findings, and thus, reinforces the additive activity interpretation.

### 2.5. Terminal Mediator Profiling Supports Coordinated Dual-Axis Cytokine and Prostaglandin Modulation

**Figure 8:**
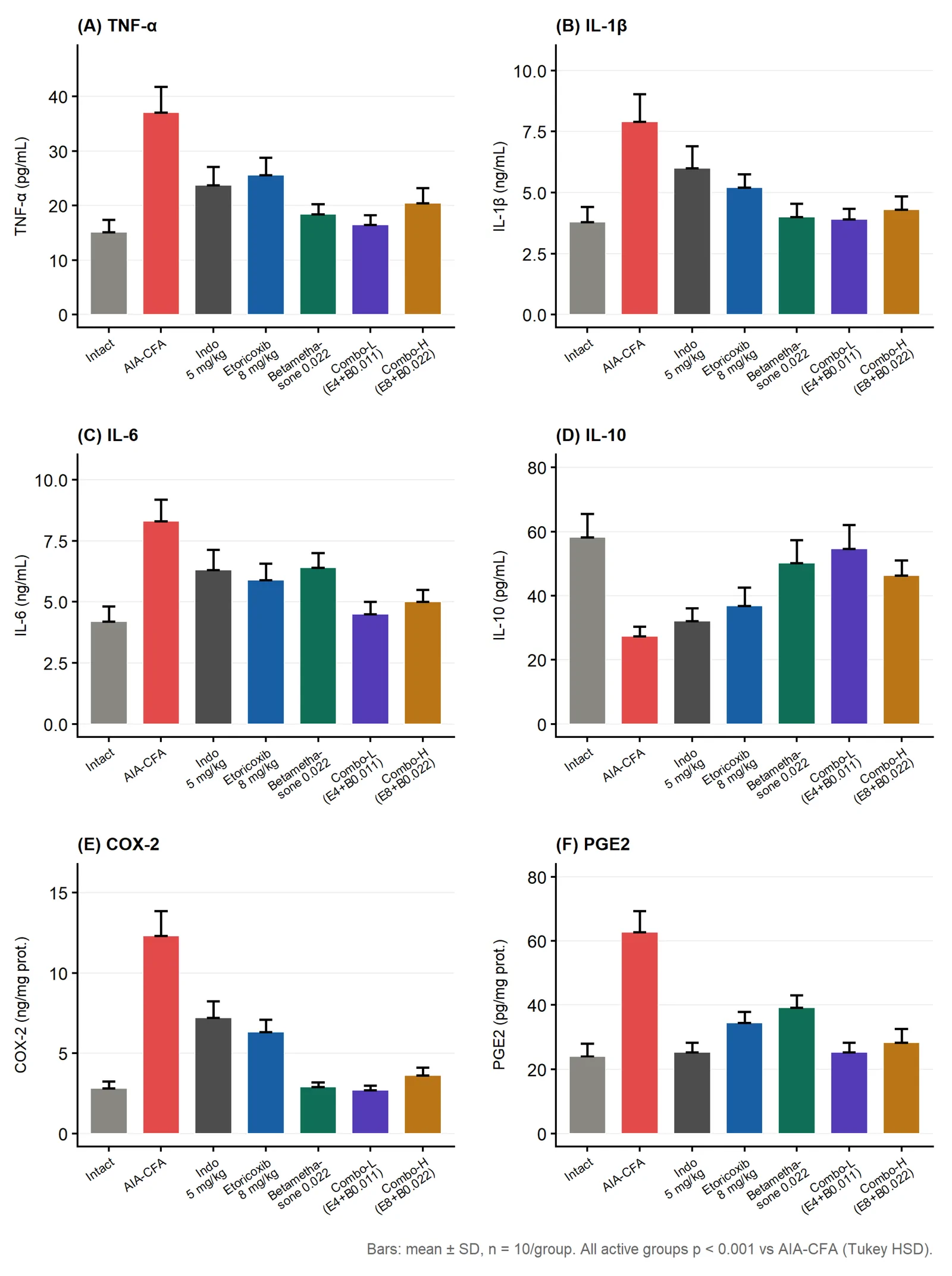
Terminal inflammatory mediator profile in inflamed paw tissue at study completion (Day 28). Cytokines and prostaglandin pathway mediators were quantified in homogenates from CFA-injected hind paw/joint-associated tissues. (**A**) TNF-α, (**B**) IL-1β, (**C**) IL-6, (**D**) IL-10 (pg/mg protein), (**E**) COX-2, and (**F**) PGE-2 (ng/mg protein). Bars represent mean ± SD (n = 10 rats/group). Versus AIA-CFA, all active treatments significantly reduced TNF-α, IL-1β, IL-6, COX-2, and PGE-2 (Tukey *p* < 0.0001 for each contrast). IL-10 increases were most pronounced for betamethasone-containing regimens: Combo-H (54.61 pg/mg protein; *p* < 0.0001 vs. AIA-CFA) approached intact control levels and did not differ significantly from the intact group (*p* = 0.8156), and Combo-L (46.31 pg/mg protein; *p* < 0.0001) also showed marked restoration; etoricoxib produced a smaller but significant increase (*p* = 0.0101), while the effect of indomethacin was not significant (*p* = 0.5576). Relative to etoricoxib, betamethasone and Combo-H reduced IL-1β (*p* = 0.0068 and *p* = 0.0026), Combo-H reduced IL-6 (*p* = 0.0003), betamethasone/Combo-L/Combo-H reduced COX-2 (all *p* < 0.0001), and indomethacin/Combo-L/Combo-H reduced PGE-2 (*p* = 0.0001, *p* = 0.022, and *p* = 0.0001, respectively). ‘E’ and ‘B’ in group labels denote etoricoxib and betamethasone dose (mg/kg), respectively; e.g., Combo-H (E8 + B0.022) = etoricoxib 8 mg/kg + betamethasone 0.022 mg/kg.

Having established that Combo-H produced superior integrated efficacy with respect to the two most differentiated behavioral endpoints (mechanical sensitivity and arthritis index), we examined the mechanisms underlying this superiority through terminal multi-analyte profiling at Day 28 (Figure 8; Table 2). The biomarker panel was selected to jointly capture two complementary anti-inflammatory axes, namely, the prostaglandin axis (COX-2, the enzyme directly targeted by etoricoxib, and its downstream product PGE-2) and the cytokine axis (TNF-α, IL-1β, and IL-6 as pro-inflammatory mediators, and IL-10 as the principal regulatory/anti-inflammatory cytokine), with the latter being the primary transcriptional target of corticosteroid action. Each terminal biomarker therefore has a direct interpretive meaning: elevation versus intact controls reflects disease-driven upregulation, and suppression by treatment toward intact levels reflects therapeutic engagement of the corresponding pathway.

The AIA-CFA disease control exhibited a markedly pro-inflammatory tissue mediator signature relative to intact animals, with approximately 2.5-fold elevations in TNF-α (37.09 ± 4.68 vs. 15.09 ± 2.31 pg/mg protein; *p* < 0.0001), IL-1β (7.90 ± 1.13 vs. 3.80 ± 0.62 pg/mg protein; *p* < 0.0001), and IL-6 (8.30 ± 0.88 vs. 4.19 ± 0.63 pg/mg protein; *p* < 0.0001), indicating substantially elevated pro-inflammatory cytokine tone in the inflamed joint tissue; marked upregulation of COX-2 (12.30 ± 1.55 vs. 2.80 ± 0.43 ng/mg protein; *p* < 0.0001) and PGE-2 (62.70 ± 6.65 vs. 24.00 ± 3.94 ng/mg protein; *p* < 0.0001), reflecting robust engagement of the prostaglandin synthesis pathway; and concurrent suppression of the regulatory cytokine IL-10 (27.40 ± 2.93 vs. 58.20 ± 7.35 pg/mg protein; *p* < 0.0001), indicating a loss of endogenous anti-inflammatory/pro-resolving tone. All active treatments significantly reduced the pro-inflammatory mediators versus AIA-CFA (Tukey vs. AIA-CFA: *p* < 0.0001 for TNF-α, IL-1β, IL-6, COX-2, and PGE-2 across all active groups), but the magnitude and pattern of modulation differed substantively between drug classes, as detailed below for the prostaglandin and cytokine axes.

At the prostaglandin axis (panels Figure 8E,F; Table 2), etoricoxib monotherapy—as expected, considering its mechanism as a selective COX-2 enzymatic inhibitor—reduced COX-2 by 48.8% and PGE-2 by 45.0%. However, betamethasone-containing regimens with betamethasone alone, 70.7% with Combo-L, and 78.0% with Combo-Hproduced substantially greater COX-2 suppression: 76.4% with betamethasone alone, 70.7% with Combo-L, and 78.0% with Combo-H. All of these values approach intact control levels with betamethasone alone, 70.7% with Combo-L, and 78.0% with Combo-H and significantly exceed etoricoxib alone (all *p* < 0.0001; Supplementary Table S2E). The deeper prostaglandin axis suppression observed for betamethasone-containing regimens reflects the corticosteroid’s additional transcriptional inhibition of COX-2 expression via NF-κB transrepression, extending beyond direct enzymatic blockade.

At the cytokine axis, etoricoxib monotherapy reduced TNF-α and IL-1β by 31.0% and 34.2%, respectively, leaving residual pro-inflammatory tone that was further attenuated by betamethasone and both combinations: betamethasone-containing regimens achieved 45.0–55.5% TNF-α reduction and 45.6–50.6% IL-1β reduction, with Combo-L restoring IL-1β and TNF-α levels to within 13.2% and 35.2% of intact controls, respectively. The IL-10 response provides the clearest pharmacological separation between drug classes: indomethacin produced only a 17.2% increase in IL-10 versus AIA-CFA (non-significant, *p* = 0.5576), etoricoxib achieved a 34.6% increase (*p* = 0.0101), while all betamethasone-containing regimens restored IL-10 by 69.0–99.3% toward intact levels (all *p* < 0.0001; fold change vs. AIA-CFA = 1.99 for Combo-H, Table 2; FC vs. indomethacin = 1.70, Table 2). This divergence is mechanistically coherent, given that corticosteroids are well-established inducers of regulatory cytokine programs through glucocorticoid receptor-mediated transcription [26], and suggests that the observed superiority of Combo-H regarding mechanical sensitivity and the arthritis index is underpinned not merely by additive prostaglandin blockade, but by a qualitatively distinct cytokine reprogramming activity that etoricoxib alone cannot provide. The cytokine axis impact—particularly IL-1β suppression and macrophage normalization—is mechanistically aligned with the NLRP3 inflammasome/IL-1β biology that drives the acute inflammatory response in gout [3,4], reinforcing the translational rationale for this drug combination in acute gout flare management.

### 2.6. Exploratory Flow Cytometry Suggests Macrophage-Associated Modulation by Betamethasone-Containing Regimens

Terminal flow cytometry was conducted on joint-associated tissues at study completion (n = 6/group, reduced subset due to tissue availability after ELISA sample collection) to evaluate the impacts of the pharmacological interventions on cellular infiltration, specifically focusing on T-lymphocytes and the macrophage/monocyte lineage (Table 2). Representative gating strategies and dot plots for the CD3+ and CD68+/CD11b+ panels are provided in Supplementary Figure S1.

Analysis of the CD3+ lymphocyte population revealed no significant overall group effect (one-way ANOVA *p* = 0.4443), with group means ranging from 3.36 ± 1.36% in the betamethasone-treated rats to 5.90 ± 1.17% in the indomethacin positive control group. These data suggest that T-lymphocyte recruitment was not a primary driver of the local inflammatory environment at this terminal stage of the disease model, consistent with the broader literature describing the chronic phase of the AIA model as predominantly macrophage-driven.

In contrast, a significant overall group effect was observed for the macrophage-associated marker panel (CD68+/CD11b+; one-way ANOVA *p* = 0.0055). The induction of adjuvant-induced arthritis led to a marked expansion of macrophage infiltration in the disease-control group (17.17 ± 6.77%) compared with the 7.33 ± 2.51% observed in intact controls (Tukey *p* = 0.0375). Although all active groups showed a downward trend, only monotherapy with betamethasone reached statistical significance in reducing these levels (to 7.47 ± 5.21%; *p* = 0.0312), effectively returning the macrophage marker percentages to intact control levels. Etoricoxib monotherapy showed minimal impact on this specific cellular readout (16.14 ± 6.06%), consistent with its lack of direct action on glucocorticoid receptor-mediated cellular reprogramming. Both combinations produced numerically large reductions versus AIA-CFA (Combo-L: 35.4%, 11.10 ± 7.00%; Combo-H: 49.0%, 8.76 ± 4.43%; fold change vs. AIA-CFA = 0.51, Table 2) that did not reach formal statistical significance in this underpowered subset, consistent with the exploratory, hypothesis-generating nature of these data. The mechanistic coherence between betamethasone’s significant macrophage normalization and the IL-10 restoration described in Section 2.5 supports the cytokine–macrophage axis as a plausible substrate for the integrated efficacy advantage observed for betamethasone-containing regimens.

## 3. Discussion

This study evaluated whether co-therapy with a selective COX-2 inhibitor (etoricoxib) and a corticosteroid (betamethasone) can improve integrated antinociceptive and anti-edema outcomes in a rat model of inflammatory arthritis. The findings are organized below into three thematic subsections—the integrated efficacy profile and the additive-vs-dose-reduction interpretation (Section 3.1), the mechanistic substrate revealed by the cytokine and prostaglandin axes (Section 3.2), and translational implications for gout flares and rheumatoid arthritis bridge therapy in clinical practice (Section 3.3)—followed by a candid statement regarding the study’s limitations (Section 3.4).

### 3.1. Integrated Efficacy and the Additive-vs.-Dose-Reduction Interpretation

Two related but distinct pharmacological concepts frame the interpretation of our data, and we define both explicitly here because they are central to how our results should be read. Additivity means that the effect of a combination is approximately equal to the sum of the effects of each individual component—that is, the drugs contribute independently without amplifying one another. Synergy means that the combined effect exceeds what would be predicted from simply adding the individual effects—that is, the drugs interact to produce a disproportionately larger response. Distinguishing between these two possibilities formally requires either isobolographic analysis under the median effect (Loewe additivity) framework or dose–response surface modeling under the Bliss independence assumption, both of which require individual dose–response curves for each agent across a range of doses [27]. Because our design tested only a single dose level per monotherapy (plus one reduced-dose combination and one full-dose combination), we cannot formally apply either framework here. Therefore, we report our findings descriptively as additive activity—the most conservative interpretation consistent with our data—and explicitly do not claim synergy.

Across the three primary efficacy endpoints (paw edema, mechanical withdrawal threshold, and clinical arthritis index), both combinations reduced disease severity versus the AIA-CFA disease control. The full-dose combination (Combo-H) consistently produced the greatest integrated effects on mechanical sensitivity and the arthritis index, exceeding both monotherapies for these endpoints. These outcomes are consistent with additive pharmacological activity for the two mechanistically complementary agents administered at their full doses, and are not interpreted as evidence of pharmacological synergy, per the definitions above. We therefore report our findings descriptively as additive activity, leaving formal interaction classification for the dedicated follow-up study outlined in Section 3.4.

By contrast, the reduced-dose combination (Combo-L)—formulated at 50% of the dose of each component—achieved integrated improvements that were statistically equivalent to those of the full-dose monotherapies across all three AUC endpoints. This pattern is consistent with a dose reduction effect, although formal demonstration of such an effect would require individual reduced-dose monotherapy arms, which were not included in the present study. A precedent for this type of finding is the work of Zimmermann and colleagues, who applied isobolographic analysis to a combination of prednisolone with a non-steroidal partner (dipyridamole) and demonstrated that their synergistic interaction enabled a 10-fold reduction in the glucocorticoid dose required for 70% TNF-α inhibition in vitro, with confirmation across rat models of lipopolysaccharide (LPS)-induced endotoxemia, delayed-type hypersensitivity, and collagen- and adjuvant-induced arthritis in vivo [27]. The relevance of that work to ours is two-fold: it establishes that a partner drug can selectively amplify glucocorticoid anti-inflammatory activity in the same disease model we used, and provides a methodological template—comprising combination dose–response matrices, isobolographic analysis, and parallel monitoring of glucocorticoid-mediated adverse effects (HPA axis suppression, osteocalcin/bone density)—that we propose to apply to the etoricoxib–betamethasone pairing in future work.

Notably, the edema endpoint showed the smallest between-treatment dynamic range of the three primary readouts, with all active treatments—alone or in combination—converging to similar AUC values. This pattern is consistent with a ceiling effect of the AIA-CFA model on paw edema once any effective anti-inflammatory mechanism is engaged. In contrast, the mechanical sensitivity (von Frey) and clinical arthritis index endpoints displayed substantially greater dynamic range and were the endpoints for which the full-dose combination outperformed both monotherapies. This dissociation between endpoints supports the use of multi-endpoint pharmacodynamic designs in preclinical arthritis pharmacology, since reliance on edema alone may underestimate the true differentiation of treatments.

### 3.2. Mechanistic Substrate: Dual-Axis Cytokine and Prostaglandin Modulation

The pattern of efficacy is consistent with two complementary anti-inflammatory programs. Selective COX-2 inhibition suppresses prostaglandin E2 (PGE-2) signaling, acting through G-protein-coupled EP1–EP4 receptors that sensitize peripheral nociceptors (EP1, EP4) and promote vascular permeability and edema (EP2, EP4) [25]. Corticosteroids, by contrast, dampen cytokine transcription via transrepression of NF-κB and AP-1 and reprogram macrophage activity toward tissue-homeostatic phenotypes [26]. This NF-κB transrepression is consequential in both clinical contexts motivating this work: in acute gout, NF-κB activation downstream of NLRP3 assembly drives the cytokine surge of the flare [3,4]; in rheumatoid arthritis, sustained NF-κB signaling in fibroblast-like synoviocytes drives the chronic synovial program [8,9,10]. The multi-analyte profile of Figure 8, quantified by ELISA for the reasons detailed in Section 4.9, provides quantitative support for both mechanisms operating in parallel in the present dataset.

At the prostaglandin axis, COX-2 protein and PGE-2 were markedly elevated in AIA-CFA animals and suppressed across all active regimens; however, the magnitude and pattern of suppression differed substantively between drug classes. Etoricoxib monotherapy, acting as a selective COX-2 enzymatic inhibitor, reduced COX-2 protein by 48.8% and PGE-2 by 45.0% versus AIA-CFA, consistent with its primary mechanism of direct enzymatic blockade; by contrast, betamethasone-containing regimens produced COX-2 suppression of 70.7–78.0% and PGE-2 suppression of 37.6% (betamethasone monotherapy), 55.0% (Combo-L), and 59.6% (Combo-H), exceeding etoricoxib alone. This finding reflects the corticosteroid’s broader transcriptional inhibition of COX-2 expression via NF-κB transrepression, extending beyond direct enzymatic blockade [14,15]. This dual mechanism explains why betamethasone monotherapy achieved prostaglandin axis effects of similar or greater magnitude than a selective COX-2 inhibitor at the doses tested.

Regarding the cytokine axis, etoricoxib monotherapy reduced TNF-α and IL-1β by 31.0% and 34.2%, respectively, leaving residual pro-inflammatory tone that was further attenuated by betamethasone and both combinations: betamethasone-containing regimens achieved 45.0–55.5% TNF-α reduction and 45.6–50.6% IL-1β reduction, with Combo-L restoring IL-1β and TNF-α levels to within 13.2% and 35.2% of intact controls, respectively. The IL-10 response provides the clearest pharmacological separation between drug classes: indomethacin produced only a 17.2% increase in IL-10 versus AIA-CFA (non-significant, *p* = 0.5576) and etoricoxib achieved a 34.6% increase (*p* = 0.0101), while the betamethasone-containing regimens restored IL-10 by 69.0–99.3% toward intact levels (all *p* < 0.0001). This divergence is mechanistically coherent: corticosteroids are well-established inducers of regulatory cytokine programs through glucocorticoid receptor-mediated transcriptional induction of IL-10 [26]—a distinct transcriptional action from the enzymatic COX-2 blockade exerted by etoricoxib, which does not directly induce IL-10 transcription. This suggests that the observed superiority of Combo-H regarding mechanical sensitivity and the arthritis index is underpinned not merely by additive prostaglandin blockade, but by a qualitatively distinct cytokine reprogramming activity that etoricoxib alone cannot provide.

The disease-specific relevance of these mediators differs somewhat between the two principal clinical scenarios motivating this work. In acute gout, IL-1β is the central effector cytokine, which is matured via NLRP3 inflammasome/caspase-1 activation downstream of monosodium urate crystal recognition, and is itself the target of approved biologic therapies (e.g., anakinra, canakinumab) in patients with contraindications to first-line agents. The deeper IL-1β suppression achieved by betamethasone-containing regimens in our data (up to 50.6% versus 34.2% for etoricoxib alone) is therefore mechanistically aligned with this disease-specific biology [3,4]. While TNF-α and IL-6 are also elevated in acute gout, they are comparatively more central to the sustained, chronic inflammatory process of rheumatoid arthritis, where they drive fibroblast-like synoviocyte activation and are direct therapeutic targets of biologic DMARDs [8]. In both conditions, IL-10 functions as a negative-feedback regulatory cytokine; its suppression during active disease and its restoration with effective therapy is generally interpreted as a marker of engaged resolution biology rather than a direct driver of joint damage, consistent with the interpretation applied here. Elevation of COX-2 and PGE-2 is common to both conditions and reflects the shared final enzymatic pathway of arachidonic acid metabolism that NSAIDs and COX-2 inhibitors directly target.

The observed impact on the cytokine axis—particularly IL-1β suppression and macrophage normalization—is mechanistically aligned with the NLRP3 inflammasome/IL-1β biology that drives the acute inflammatory response in gout [3,4], reinforcing the translational rationale for this drug combination in acute gout flare management.

### 3.3. Translational Implications for Clinical Practice

The following discussion is explicitly framed as hypothesis-generating; the preclinical findings reported here provide mechanistic plausibility for the etoricoxib–betamethasone combination, but do not constitute clinical proof of efficacy and are not intended to substitute for the evidence being generated in ongoing Phase III trials (NCT06863701; NCT07328022). The clinical relevance of these findings derives from the complementarity of the two mechanisms under study. In current practice, NSAIDs and corticosteroids are already co-administered in acute inflammatory settings, including gout flares and short-course corticosteroid bridge therapy in rheumatoid arthritis (RA)—namely, time-limited (typically 2–6 month) corticosteroid co-administration alongside a DMARD while the DMARD’s full effect develops—on the basis of guideline support rather than formal dose optimization data [7,13]. The present preclinical data provide a quantified mechanistic rationale for this widespread clinical practice: across the six terminal mediators assessed, betamethasone-containing regimens—including the reduced-dose treatment (Combo-L)—produced coordinated shifts in the prostaglandin axis (COX-2 and PGE2) and the cytokine axis (TNF-α, IL-1β, IL-6, and IL-10) that neither etoricoxib monotherapy nor indomethacin achieved alone. In particular, in the cytokine axis, indomethacin matched Combo-H for PGE-2 suppression (FC vs. indomethacin = 1.00, Table 2) but produced only a non-significant 17.2% increase in IL-10 versus AIA-CFA compared with the 70-fold increase (FC = 1.70 vs. indomethacin, Table 2) achieved by Combo-H, which restored IL-10 to within 6.2% of intact control levels.

This rationale is particularly relevant for the clinical management of acute gout flares, where the NLRP3 inflammasome–IL-1β axis is the central driver of joint inflammation following monosodium urate crystal deposition [3,4]. The deeper IL-1β suppression observed with Combo-H (50.6% reduction vs. AIA-CFA, restoring IL-1β to within 2.6% of intact controls) compared with etoricoxib monotherapy (34.2% reduction) demonstrates the mechanistic plausibility of an etoricoxib–betamethasone fixed-dose combination for acute gout therapy. A Phase III clinical trial comparing an etoricoxib/betamethasone fixed-dose combination (90 mg/0.25 mg once daily) versus etoricoxib alone in patients with acute gouty arthritis (NCT06863701), thus directly evaluating this hypothesis, is currently recruiting [28]. A parallel Phase III trial evaluating the same fixed-dose combination in acute bursitis, tendinitis, and synovitis (also vs. etoricoxib monotherapy) is also in progress (NCT07328022) [29]. The present preclinical study thus provides timely mechanistic evidence in alignment with these ongoing clinical investigations. Furthermore, comprehensive pharmacokinetic and biodistribution characterization of the combination—building on the individually established human pharmacokinetic (PK) profiles of etoricoxib and betamethasone, together with dedicated safety pharmacology and repeat-dose toxicology—are being generated separately as part of the Investigational New Drug (IND)-enabling regulatory package supporting this clinical program; however, these efforts fall outside the pharmacodynamic scope of the present manuscript.

A frequently raised comparator question is why an etoricoxib–betamethasone combination would be developed when, on several endpoints, Combo-H performed similarly to indomethacin. The three following considerations address this point: (i) safety profile—indomethacin is a nonselective COX-1/COX-2 inhibitor with substantial gastrointestinal, renal, and cardiovascular liabilities that restrict its use in patients with peptic ulcer history, anticoagulation, or cardiometabolic comorbidity, whereas etoricoxib’s selective COX-2 inhibition confers materially improved gastrointestinal tolerability [18]; (ii) dose reduction rationale—Combo-L achieved efficacy equivalent to full-dose monotherapies at 50% of each component dose, thus suggesting a dose-sparing strategy with no comparable analog available for indomethacin monotherapy, one that would be expected to lower cumulative NSAID and corticosteroid exposure in the context of short-course use; and (iii) mechanism-of-action diversity—the dual-axis cytokine and prostaglandin suppression achieved by betamethasone-containing regimens, particularly the near-complete IL-10 restoration that neither indomethacin nor etoricoxib alone can achieve, provides a qualitatively distinct anti-inflammatory profile with potential relevance in disease states where regulatory cytokine engagement is therapeutically desirable. The clinical comparator against which this combination is being tested in the ongoing Phase III program is etoricoxib monotherapy rather than indomethacin, reflecting etoricoxib’s more favorable baseline safety profile as an appropriate reference standard.

In the rheumatoid arthritis bridge-therapy setting, the clinical evidence base for combining NSAIDs and corticosteroids has matured substantially and directly informs our Combo-L findings. The CareRA trial showed that methotrexate with step-down glucocorticoid bridging (COBRA-Slim, from the COmbinatie therapie Bij Reumatoïde Artritis [COBRA] regimen) produced sustained 5-year disease control non-inferior to more aggressive csDMARD combinations, with reduced chronic NSAID use [30]. The GLORIA (Glucocorticoid LOw-dose in RheumatoId Arthritis) trial extended this to elderly RA patients, showing that 5 mg/day prednisolone for 2 years provided consistent benefit in disease activity (DAS28) without excess age-related adverse events [31]. The BARFOT (Better Anti-Rheumatic FarmacOTherapy; Swedish early-RA cohort)-derived comparison by Krause et al. found that low-dose (10 mg) prednisolone bridging gave outcomes comparable to high-dose (60 mg) bridging in early RA [32], directly supporting the dose-minimization principle underlying our Combo-L design. Likewise, a post-hoc analysis by Kvien and colleagues found that etoricoxib produced additional pain relief in RA patients already on corticosteroids, with consistent effect sizes across background therapy subgroups [33]. Collectively, this evidence indicates that a low-dose etoricoxib–betamethasone combination—as exemplified by our Combo-L—aligns with the guideline-driven trend toward minimum-effective glucocorticoid exposure in short-course inflammatory disease management.

A potential clinical advantage of administering reduced doses of both agents (again, exemplified by the Combo-L profile in our study) is the opportunity to mitigate adverse event risk when short-course therapy is required. Within this study’s 28-day preclinical design, no overt safety signals were dominant; however, a formal safety interpretation is constrained by the study duration, species, and the limited safety endpoints assessed, as detailed in Section 3.4. Translational emphasis should remain on short-course, acute-episode scenarios where the risk–benefit balance of corticosteroid co-administration is most favorable, and where dose minimization is most clinically relevant. Head-to-head preclinical or clinical comparison against other RA disease-modifying strategies (e.g., biologic or targeted synthetic DMARDs) was outside the scope of the present short-course pharmacodynamic study, as such agents are designed to achieve long-term disease modification over a materially different treatment horizon (e.g., weeks to months until onset of effect) than the acute symptom control window evaluated here.

Taken together, the presented preclinical data provide, to our knowledge, the first head-to-head pharmacodynamic characterization of the etoricoxib + betamethasone fixed-dose combination in a standardized model of inflammatory arthritis, addressing a gap explicitly identified in the most recent systematic review of NSAID and corticosteroid combination therapy in RA, namely, that no trial or preclinical study has directly compared a fixed NSAID–corticosteroid combination against either monotherapy with quantitative dose–response data [17]. Validation of the mechanistic hypotheses generated here against human patient samples (e.g., synovial fluid or serum cytokine profiling before and after treatment) is an appropriate next step for parallel clinical trials [28,29] rather than for the present preclinical pharmacodynamic study, consistent with the conventional and regulatory separation between preclinical hypothesis generation and clinical biomarker validation.

### 3.4. Limitations

Key limitations include: (i) the absence of individual reduced-dose monotherapy groups (etoricoxib 4 mg/kg alone; betamethasone 0.011 mg/kg alone) precludes formal dose-sparing conclusions and isobolographic interpretation—the primary limitation of this work, which a complete dose-optimization design (a 4 × 4 dose–response matrix with isobolographic analysis at multiple effect levels) would address; (ii) the absence of a formal synergy framework, as defined in Section 3.1; (iii) terminal biomarkers measured at a single time point (Day 28) rather than as time-resolved kinetics; (iv) exploratory immune profiling that warrants confirmation with pre-specified panels and adequate power, particularly for the macrophage compartment (CD68+/CD11b+), which showed a numerically large reduction (49.0% for Combo-H) that did not reach significance in this underpowered subset and would benefit from complementary histopathological and immunohistochemical confirmation in a follow-up study; (v) a minor procedural asymmetry, as the intact group received only a single saline injection rather than the CFA booster given to arthritic groups; (vi) the AIA-CFA model reflects chronic polyarticular inflammation rather than the acute, monosodium urate (MSU) crystal-driven synovitis most directly relevant to gout flares, for which translational confirmation in an MSU model is an important next step; (vii) only male rats were used, consistent with standard practice in this model but restricting extrapolation to females; future work should incorporate both sexes per SAGER guidance; and (viii) the 28-day duration was deliberately matched to the intended short-course clinical use of this combination; longer-duration studies would be required for chronic indications.

Future work incorporating individual reduced-dose monotherapy arms, dose–response surface modeling, acute flare models (e.g., MSU-induced synovitis), time-resolved mediator sampling, histopathological and immunohistochemical confirmation, and dedicated safety endpoints (e.g., HPA axis function, hematology, hepatorenal panels, and body weight trajectory) will be required to confirm the most clinically relevant regimen and to formally establish any dose reduction benefit of the combination considered here.

## 4. Materials and Methods

### 4.1. Study Design and Reporting

This randomized, blinded, parallel group study assessed the anti-inflammatory and antinociceptive effects of etoricoxib, betamethasone, and their combinations in the complete Freund’s adjuvant (CFA) model of adjuvant-induced arthritis (AIA) in rats. Reporting follows the ARRIVE 2.0 guidelines [34], which provide the internationally recognized standard for reporting in vivo animal research to maximize transparency, reduce reporting bias, and support the reproducibility of preclinical pharmacological findings.

Treatments were coded such that the personnel performing the outcome assessments were blinded to group allocation.

### 4.2. Animals and Housing

Seventy male Wistar rats (Rattus norvegicus; ~10 weeks old; 250–300 g at study start) were acclimated for 7 days prior to procedures. Animals were housed under controlled environmental conditions (22 ± 2 °C; 12 h light/dark cycle) with ad libitum access to standard chow and water. The Wistar strain and male sex were selected on methodological grounds: the AIA-CFA model has been extensively pharmacologically characterized in this strain, enabling direct comparison of effect sizes with the published literature, and male animals were used to avoid the confounding influence of estrous-cycle-related hormonal variability on pain behavior assessment, consistent with standard practice in preclinical inflammatory pain pharmacology. This choice is acknowledged as a limitation with respect to generalizability of the results to female animals (Section 3.4).

### 4.3. Ethical Approval

All procedures were conducted in accordance with Mexican regulations for the care and use of laboratory animals (NOM-062-ZOO-1999) and the International Association for the Study of Pain (IASP) guidelines for pain research in animals, and were approved by the institutional ethics/animal care committee (Centro Regional de Biociencias, UASLP; approval code: JUN-2025/001).

### 4.4. Induction of Adjuvant-Induced Arthritis

Arthritis was induced on Day 0 via intradermal administration of 0.1 mL CFA (Mycobacterium tuberculosis H37Ra, 10 mg/mL in mineral oil, Sigma-Aldrich, St. Louis, MO, USA) into the plantar surface of the hind paw. A booster immunization (0.05 mL CFA) was administered intradermally at the base of the tail on Day 4. The intact control group received a single intradermal injection of sterile saline (0.1 mL) on Day 0, administered into the plantar surface of the ipsilateral hind paw (the same anatomical site as the primary CFA inoculation). This injection corresponds to the primary CFA injection site and serves as the procedural control for the needle penetration and local tissue disturbance associated with the primary CFA challenge; no second injection was administered to intact controls, as the booster is specific to the arthritis induction protocol and lacks a physiologically equivalent saline control. Animals that did not develop clinical signs consistent with arthritis (paw edema/erythema) were excluded as per the protocol.

### 4.5. Treatments and Dose Selection

Doses were selected based on clinically relevant human doses and translated to rats using body surface area-based allometric scaling [35], then refined based on tolerability considerations. Briefly, human clinical doses (etoricoxib 90 mg/day; betamethasone 0.25 mg/day, consistent with the corresponding fixed-dose combination formulation; indomethacin 50 mg three times daily as an established positive control regimen) were converted to rat-equivalent doses using the body surface area normalization method recommended by the U.S. Food and Drug Administration, applying a human-to-rat conversion factor of 6.2 (i.e., rat dose in mg/kg = human dose in mg/kg × 6.2) [35]. The resulting rat doses were then cross-checked, and were found to be consistent with doses reported in prior preclinical work using the AIA-CFA model. All treatments were administered once daily by oral gavage (8:00–9:00 AM) from Day 4 through Day 28. Formulations were prepared in 0.5% carboxymethylcellulose (CMC) as vehicle (including betamethasone in 0.5% CMC); dosing volume was 2 mL/kg. For combination groups, both drugs were administered together in the same gavage dose.

Animals were allocated to seven groups (n = 10/group): Intact (control), AIA-CFA (disease control), indomethacin 5 mg/kg (positive control), etoricoxib 8 mg/kg, betamethasone 0.022 mg/kg, low-dose combination (Combo-L, etoricoxib 4 mg/kg + betamethasone 0.011 mg/kg), and high-dose combination (Combo-H, etoricoxib 8 mg/kg + betamethasone 0.022 mg/kg).

### 4.6. Paw Edema (Plethysmometry)

Hind paw volume (ipsilateral/CFA-injected paw) was measured via water displacement plethysmometry (Model 7140, Ugo Basile, Comerio, Italy) at Day 0 (pre-dose), Day 0 + 3 h, Day 1 + 3 h, and on Days 2, 4, 5, 7, 9, 11, 14, 21, and 28. The Day 0 + 3 h and Day 1 + 3 h measurements were performed to capture the acute inflammatory peak of the CFA response (approximately 2–4 h post-injection), prior to the model’s evolution into its chronic phase, thereby documenting the full time course of the inflammatory response from onset. Paw volume was determined as the means of two consecutive displacement measurements obtained by the same operator at each assessment time point, performed immediately after von Frey testing. Paw edema is summarized as time course profiles and area under the curve (AUC) values across the observation period using the trapezoidal rule. The AUC, a standard approach in preclinical inflammation pharmacology, was selected as the integrated pharmacodynamic readout because it captures total disease burden across the 28-day observation period independent of variability at a single time point.

### 4.7. Mechanical Allodynia (Von Frey)

Von Frey mechanical withdrawal thresholds quantify inflammation-driven mechanical hypersensitivity (allodynia), a primary behavioral correlate of joint pain in the AIA-CFA model and a widely used translational endpoint in preclinical arthritis pharmacology. Mechanical withdrawal thresholds were assessed using calibrated von Frey filaments (Aesthesio^®^; Ugo Basile, Comerio, Italy) applied to the plantar surface of the ipsilateral hind paw, following an established approach for quantifying mechanical hypersensitivity in rodents [36]. Animals were habituated to the testing chambers on a wire mesh platform prior to assessment. Von Frey testing was performed prior to plethysmometry at each session, avoiding any potential sensitization caused by the water displacement procedures. The withdrawal threshold (g) was defined as the minimum filament force eliciting a brisk paw withdrawal response and was recorded at the same time points as paw volume.

### 4.8. Clinical Arthritis Score

Arthritis severity was assessed in a blinded manner using an ordinal clinical score (0–3) based on erythema and edema of the paws: 0 = normal; 1 = mild edema/erythema; 2 = moderate swelling and redness; 3 = severe swelling and deformity. Scores were recorded at the same time points as paw volume measurements and summarized as time course and AUC values.

### 4.9. Enzyme-Linked Immunosorbent Assay (ELISA)

At study termination (Day 28), animals were deeply anesthetized via intraperitoneal injection of xylazine (8 mg/kg) and ketamine (70 mg/kg), followed by euthanasia via pentobarbital sodium overdose (800 mg/kg, i.p.). Joint tissues were harvested and homogenized in phosphate-buffered saline (PBS; pH 7.4) supplemented with a protease inhibitor cocktail and 0.1% Triton X-100 (ratio 1:10). Homogenates were centrifuged at 12,000× *g* for 20 min at 4 °C. Total protein content in tissue homogenate supernatants was quantified using a Pierce™ BCA Protein Assay Kit (Thermo Fisher Scientific, Waltham, MA, USA) according to the manufacturer’s instructions, with bovine serum albumin (BSA) as the protein standard. The concentrations of inflammatory mediators in the supernatants were quantified using the following commercial sandwich ELISA kits (Thermo Fisher Scientific/Invitrogen, Waltham, MA, USA): TNF-alpha, Rat TNF-alpha ELISA Kit (Cat. KRC3011); IL-1beta, Rat IL-1 beta ELISA Kit (Cat. KRC0011); IL-6, Rat IL-6 ELISA Kit (Cat. KRC0061); IL-10, Rat IL-10 ELISA Kit (Cat. KRC0101); PGE2, Prostaglandin E2 ELISA Kit (Cat. EMSPGE2); and COX-2, Rat COX-2 ELISA Kit (Cat. ERCX2). Homogenate supernatants were diluted in the manufacturer-supplied assay buffer at ratios of 1:2 to 1:10 depending on the analyte, according to kit-specific validated ranges. Primary capture antibodies were pre-coated on the supplied 96-well plates; samples and standards were incubated for 2 h at room temperature with gentle agitation, followed by a biotinylated detection antibody (1 h incubation) and a streptavidin–horseradish peroxidase (HRP) conjugate. Blocking was performed using the manufacturer’s proprietary blocking buffer included in the respective kit. Detection was performed via 3,3′,5,5′-tetramethylbenzidine (TMB) substrate development (15 min in the dark) followed by a stop solution. Absorbance was measured at 450 nm using a Multiskan™ FC microplate reader (Thermo Fisher Scientific/Invitrogen, Waltham, MA, USA). The results were normalized to total protein content and are expressed as pg/mg protein (cytokines) or ng/mg protein (COX-2, PGE2).

### 4.10. Flow Cytometry

Single-cell suspensions from joint-associated tissues were generated via mechanical dissociation and enzymatic digestion, followed by filtration through 70 μm strainers. Approximately 1 × 10^6^ cells per sample were loaded for staining, and at least 100,000 events were acquired per sample for analysis. Cells were washed and stained with the following fluorochrome-conjugated antibodies (BD Biosciences, San Jose, CA, USA): Macrophage panel, Anti-CD11b (Cat. 562102) and Anti-CD68 (Cat. 566311); and T-lymphocyte panel, Anti-CD3 (Cat. 554832). Following incubation for 30 min at 4 °C in the dark, the cells were fixed in 1% formaldehyde prior to acquisition on a multicolor flow cytometer. Data were analyzed using standard gating strategies with blinded sample IDs. Representative gating strategies and dot plots are provided in Supplementary Figure S1.

### 4.11. Statistical Analysis

Data are presented as mean ± SEM for time course profiles and mean ± SD for integrated (AUC) and terminal endpoints. Normality was assessed using the Shapiro–Wilk test. Group comparisons for endpoints (AUC metrics, terminal biomarkers) at a single time point were performed using one-way ANOVA followed by Tukey’s post hoc test for all pairwise comparisons; this approach was selected because it controls the family-wise error rate across the multiple pairwise contrasts of interest (i.e., each active group vs. AIA-CFA, and combinations vs. monotherapies). Time course data were analyzed using linear mixed-effects models with treatment, time, and their interaction as fixed effects and subject as a random effect, which was considered appropriate given the repeated, within-subject structure of the longitudinal behavioral data. Receiver operating characteristic (ROC) analysis was not performed, as the present study addresses a pharmacodynamic intervention question (regarding the magnitude and significance of treatment effects) rather than a diagnostic classification question (e.g., discrimination between disease and healthy states using a biomarker threshold), for which ROC analysis would be the appropriate tool. All analyses were conducted using StatsDirect (version 4.0; StatsDirect Ltd., Birkenhead, UK), and *p* < 0.05 was considered statistically significant. Complete pairwise Tukey comparison tables for all endpoints are provided in Supplementary Tables S1 and S2.

## 5. Conclusions

In the AIA-CFA rat model, the etoricoxib–betamethasone combinations produced sustained improvements in paw edema and mechanical hypersensitivity relative to disease controls. In particular, the full-dose combination (Combo-H) produced integrated outcomes exceeding those of individual monotherapies for mechanical sensitivity and arthritis severity, consistent with additive pharmacological activity for two mechanistically complementary agents. The low-dose combination (Combo-L) achieved integrated improvements statistically equivalent to those of full-dose monotherapies for all three primary endpoints (paw edema, mechanical withdrawal threshold, and clinical arthritis index), a pattern that is consistent with—but does not formally establish—a dose reduction effect. Concordant shifts in prostaglandin-linked mediators (COX-2, PGE-2) and cytokine-linked mediators (TNF-α, IL-1β, IL-6, IL-10), alongside exploratory macrophage-associated signals, support a plausible cytokine–macrophage axis as the mechanistic substrate for the integrated efficacy of the assessed co-therapy.

To the best of our knowledge, these preclinical data represent the first published head-to-head pharmacodynamic characterization of the etoricoxib + betamethasone combination—as a specific, clinically advancing pairing rather than an arbitrary combinatorial selection—in a standardized model of inflammatory arthritis, with parallel behavioral, biochemical, and exploratory immunological endpoints. This addresses an evidence gap explicitly identified in the most recent systematic review and network meta-analysis of NSAID–corticosteroid combination therapies in rheumatoid arthritis, which found the existing trial literature insufficient to support quantitative comparison of a fixed combination against either monotherapy [17]. From a translational perspective, these findings provide a quantified mechanistic rationale for combining a selective COX-2 inhibitor with a corticosteroid for the management of acute inflammatory flares—a practice that is already endorsed in current rheumatology and gout guidelines [5,7,16] but which lacks dedicated clinical dose optimization data [17].

The mechanistic alignment between the IL-1β-suppressing activity of betamethasone-containing regimens documented here and the NLRP3 inflammasome–IL-1β biology that drives acute gout flares [3,4] reinforces the translational plausibility of the etoricoxib–betamethasone fixed-dose combination currently under Phase III evaluation in the context of acute gouty arthritis (NCT06863701) [28] and acute bursitis, tendinitis, and synovitis (NCT07328022) [29]. However, confirmation of the dose reduction signal will require individual reduced-dose monotherapy groups and formal isobolographic analysis. Looking forward, prospective preclinical work incorporating dose–response surface modeling, acute flare models (e.g., MSU-induced synovitis), time-resolved mediator sampling, and dedicated safety endpoints will be necessary to establish the minimum effective combination dose. Together with the growing clinical evidence base for low-dose glucocorticoid bridge therapy in rheumatoid arthritis [30,31,32], this study’s findings support the continued evaluation of short-course etoricoxib–betamethasone regimens for acute inflammatory conditions where rapid symptom control and minimization of cumulative exposure are both clinically essential.

## Figures and Tables

**Table 1:** Integrated efficacy endpoints (AUC) for paw edema (Δ paw volume), mechanical withdrawal threshold (von Frey), and arthritis index across the 28-day study, with fold change (FC) comparisons versus AIA-CFA and versus indomethacin. Values are mean ± SD; n = 10 per group.

Group	Edema AUC (mL·Day)	FC vs. AIA-CFA	FC vs. Indo	von Frey AUC (g·Day)	FC vs. AIA-CFA	FC vs. Indo	Arthritis AUC (Score·Day)	FC vs. AIA-CFA	FC vs. Indo
**Intact**	2.23 ± 8.33	0.14	—	1431.73 ± 184.07	3.09	—	0.00 ± 0.00	0.00	—
**AIA-CFA**	16.37 ± 3.32	1.00	—	462.86 ± 56.86	1.00	—	43.74 ± 9.51	1.00	—
**Indomethacin**	7.45 ± 2.26	0.46	—	1131.12 ± 273.04	2.44	—	19.93 ± 8.01	0.46	—
**Etoricoxib**	8.59 ± 3.55	0.52	1.15	937.48 ± 223.80	2.03	0.83	30.77 ± 5.55	0.70	1.54
**Betamethasone**	8.89 ± 1.82	0.54	1.19	884.15 ± 226.34	1.91	0.78	34.04 ± 8.08	0.78	1.71
**Combo-L**	7.16 ± 2.06	0.44	0.96	1028.86 ± 276.61	2.22	0.91	27.56 ± 8.12	0.63	1.38
**Combo-H**	5.09 ± 1.33	0.31	0.68	1252.01 ± 288.89	2.70	1.11	18.23 ± 11.29	0.42	0.91

Combo-L: etoricoxib 4 mg/kg + betamethasone 0.011 mg/kg; Combo-H: etoricoxib 8 mg/kg + betamethasone 0.022 mg/kg. FC = fold change. Complete pairwise Tukey contrast matrices for all three endpoints are provided in Supplementary Table S1.

**Table 2:** Inflammatory biomarkers and inflammatory cell infiltration in the AIA-CFA model, with fold change (FC) of Combo-H versus AIA-CFA. Values are mean ± SD (n = 10 rats/group for biomarkers and n = 6 rats/group for inflammatory cell infiltration). *p*-values in parentheses represent Tukey-adjusted post hoc comparisons of each treatment group versus the AIA-CFA disease control.

Endpoint	Intact	AIA-CFA	Indomethacin (*p* vs. AIA-CFA)	Etoricoxib (*p* vs. AIA-CFA)	Betamethasone (*p* vs. AIA-CFA)	Combo-L (*p* vs. AIA-CFA)	Combo-H (*p* vs. AIA-CFA)	FC Combo-H vs. AIA-CFA
**TNF-α** **(pg/mg protein)**	15.09 ± 2.31	37.09 ± 4.68	23.69 ± 3.39 *(<0.0001)*	25.60 ± 3.17 *(<0.0001)*	18.40 ± 1.86 *(<0.0001)*	20.40 ± 2.81 *(<0.0001)*	16.50 ± 1.77 *(<0.0001)*	0.44
**IL-1β** **(pg/mg protein)**	3.80 ± 0.62	7.90 ± 1.13	6.00 ± 0.91 *(<0.0001)*	5.20 ± 0.56 *(<0.0001)*	4.00 ± 0.55 *(<0.0001)*	4.30 ± 0.55 *(<0.0001)*	3.90 ± 0.45 *(<0.0001)*	0.49
**IL-6** **(pg/mg protein)**	4.19 ± 0.63	8.30 ± 0.88	6.30 ± 0.82 *(<0.0001)*	5.90 ± 0.67 *(<0.0001)*	6.40 ± 0.60 *(<0.0001)*	5.00 ± 0.48 *(<0.0001)*	4.50 ± 0.50 *(<0.0001)*	0.54
**IL-10** **(pg/mg protein)**	58.20 ± 7.35	27.40 ± 2.93	32.10 ± 3.97 *(N.S.)*	36.88 ± 5.65 *(0.0101)*	50.19 ± 7.18 *(<0.0001)*	46.31 ± 4.67 *(<0.0001)*	54.61 ± 7.54 *(<0.0001)*	1.99
**COX-2** **(ng/mg protein)**	2.80 ± 0.43	12.30 ± 1.55	7.20 ± 1.03 *(<0.0001)*	6.30 ± 0.78 *(<0.0001)*	2.90 ± 0.29 *(<0.0001)*	3.60 ± 0.50 *(<0.0001)*	2.70 ± 0.29 *(<0.0001)*	0.22
**PGE-2** **(ng/mg protein)**	24.00 ± 3.94	62.70 ± 6.65	25.30 ± 2.99 *(<0.0001)*	34.50 ± 3.42 *(<0.0001)*	39.10 ± 3.95 *(<0.0001)*	28.20 ± 4.34 *(<0.0001)*	25.30 ± 3.01 *(<0.0001)*	0.40
**CD3+ lymphocytes (%)**	4.60 ± 0.61	4.70 ± 0.97	5.90 ± 1.17 *(N.S.)*	4.70 ± 0.87 *(N.S.)*	3.36 ± 1.36 *(N.S.)*	4.48 ± 2.55 *(N.S.)*	4.52 ± 1.97 *(N.S.)*	0.96
**CD68+/CD11b+ macrophages (%)**	7.33 ± 2.51	17.17 ± 6.77	9.61 ± 3.56 *(N.S.)*	16.14 ± 6.06 *(N.S.)*	7.47 ± 5.21 *(0.0312)*	11.10 ± 7.00 *(N.S.)*	8.76 ± 4.43 *(N.S.)*	0.51

Abbreviations: AIA, adjuvant-induced arthritis; CFA, complete Freund’s adjuvant; COX-2, cyclooxygenase-2; PGE-2, prostaglandin E2; N.S., not significant; FC, fold change. Combo-L: etoricoxib 4 mg/kg + betamethasone 0.011 mg/kg; Combo-H: etoricoxib 8 mg/kg + betamethasone 0.022 mg/kg. Complete pairwise Tukey contrast matrices for all biomarkers are provided in Supplementary Tables S2A–F.

## Data Availability

Data is contained within the article.

## Supplementary Materials

The following supporting information can be downloaded at: https://www.mdpi.com/article/10.3390/ph19081235/s1, Supplementary Table S1. Complete Tukey HSD pairwise comparison matrix for integrated efficacy endpoints (AUC); Supplementary Table S2. Complete Tukey’s HSD pairwise comparison matrix for inflammatory biomarkers (ELISA); Supplementary Figure S1. Representative flow cytometry gating strategies and dot plots for CD3+ and CD68+/CD11b+ panels.

## Abbreviations

The following abbreviations are used in this manuscript, listed alphabetically for reference.

**Abbreviation**	**Full Term**
**AIA**	Adjuvant-induced arthritis
**AP-1**	Activator protein-1
**ARRIVE**	Animal Research: Reporting of In Vivo Experiments
**AUC**	Area under the curve
**BARFOT**	Better Anti-Rheumatic FarmacOTherapy (Swedish early-RA cohort)
**BSA**	Bovine serum albumin
**CFA**	Complete Freund’s adjuvant
**COBRA**	COmbinatie therapie Bij Reumatoïde Artritis (Combination therapy in Rheumatoid Arthritis)
**COX-2**	Cyclooxygenase-2
**CMC**	Carboxymethylcellulose
**DAS28**	Disease Activity Score (28 joints)
**DMARD**	Disease-modifying antirheumatic drug
**ELISA**	Enzyme-linked immunosorbent assay
**EP**	Prostaglandin E receptor
**FC**	Fold change
**GLORIA**	Glucocorticoid LOw-dose in RheumatoId Arthritis trial
**HPA**	Hypothalamic–pituitary–adrenal
**HRP**	Horseradish peroxidase
**IASP**	International Association for the Study of Pain
**IHC**	Immunohistochemistry
**IL**	Interleukin
**IND**	Investigational New Drug
**LPS**	Lipopolysaccharide
**MSU**	Monosodium urate
**NF-κB**	Nuclear factor kappa-light-chain-enhancer of activated B cells
**NLRP3**	NLR family pyrin domain containing 3
**NSAID**	Non-steroidal anti-inflammatory drug
**PBS**	Phosphate-buffered saline
**PGE-2/PGE2**	Prostaglandin E2
**qRT-PCR**	Quantitative reverse-transcription polymerase chain reaction
**RA**	Rheumatoid arthritis
**RANKL**	Receptor activator of nuclear factor kappa-B ligand
**ROC**	Receiver operating characteristic
**SAGER**	Sex and Gender Equity in Research (reporting guideline)
**SEM**	Standard error of the mean
**SD**	Standard deviation
**TMB**	3,3′,5,5′-Tetramethylbenzidine
**TNF-α**	Tumor necrosis factor alpha
