# Supplementary material for: Etoricoxib–Betamethasone Combination Attenuates Inflammatory Nociception and Edema in Adjuvant-Induced Arthritis via Cytokine and Macrophage Axis Modulation
Source: Pharmaceuticals (Basel). 2026 Aug 6;19(8):1235. doi: 10.3390/ph19081235 (PMC13516479; doi:10.3390/ph19081235)
Supplement: Supplementary file 1 [file pharmaceuticals-19-01235-s001.zip › pharmaceuticals-4364694-supplementary.pdf]

Manuscript ID: pharmaceuticals-4364694

Title

Etoricoxib–betamethasone combination attenuates inflammatory nociception and edema in adjuvant-induced arthritis via cytokine and macrophage axis modulation

Supplementary Table S1 — Complete Tukey HSD Pairwise Comparison Matrix for Integrated Efficacy Endpoints (AUC)

AUC Edema (mL·day)

Group A	Group B	p-adj	Significance
AIA-CFA	Intact	<0.0001	***
AIA-CFA	Indomethacin	<0.0001	***
AIA-CFA	Etoricoxib	0.0007	***
AIA-CFA	Betamethasone	0.0013	**
AIA-CFA	Combo-H	<0.0001	***
AIA-CFA	Combo-L	<0.0001	***
Etoricoxib	Combo-H	0.4021	ns
Etoricoxib	Combo-L	0.9804	ns
Betamethasone	Combo-H	0.3052	ns
Betamethasone	Combo-L	0.9509	ns
Combo-H	Combo-L	0.8900	ns
Indomethacin	Etoricoxib	0.9941	ns
Indomethacin	Betamethasone	0.9801	ns
Indomethacin	Combo-H	0.8137	ns
Indomethacin	Combo-L	1.0000	ns

AUC von Frey (g·day)

Group A	Group B	p-adj	Significance
AIA-CFA	Intact	<0.0001	***
AIA-CFA	Indomethacin	<0.0001	***
AIA-CFA	Etoricoxib	0.0004	***
AIA-CFA	Betamethasone	0.0024	**

Group A	Group B	p-adj	Significance
AIA-CFA	Combo-H	<0.0001	***
AIA-CFA	Combo-L	<0.0001	***
Etoricoxib	Combo-H	0.0498	*
Etoricoxib	Combo-L	0.9735	ns
Betamethasone	Combo-H	0.0119	*
Betamethasone	Combo-L	0.7988	ns
Combo-H	Combo-L	0.3308	ns
Indomethacin	Etoricoxib	0.5036	ns
Indomethacin	Betamethasone	0.2190	ns
Indomethacin	Combo-H	0.9023	ns
Indomethacin	Combo-L	0.9542	ns

AUC Arthritis Index (score·day)

Group A	Group B	p-adj	Significance
AIA-CFA	Intact	<0.0001	***
AIA-CFA	Indomethacin	<0.0001	***
AIA-CFA	Etoricoxib	0.0094	**
AIA-CFA	Betamethasone	0.1097	ns
AIA-CFA	Combo-H	<0.0001	***
AIA-CFA	Combo-L	0.0005	***
Etoricoxib	Combo-H	0.0135	*
Etoricoxib	Combo-L	0.9712	ns
Betamethasone	Combo-H	0.0007	***
Betamethasone	Combo-L	0.5402	ns
Combo-H	Combo-L	0.1375	ns
Indomethacin	Etoricoxib	0.0502	ns

Group A	Group B	p-adj	Significance
Indomethacin	Betamethasone	0.0034	**
Indomethacin	Combo-H	0.9991	ns
Indomethacin	Combo-L	0.3404	ns

* p < 0.05; ** p < 0.01; *** p < 0.001; ns = not significant. All comparisons by one-way ANOVA followed by Tukey HSD post-hoc test. n = 10/group. AUC calculated by trapezoidal rule from individual animal data.

Supplementary Table S2 — Complete Tukey HSD Pairwise Comparison Matrix for Inflammatory Biomarkers (ELISA)

S2A. TNF-α (pg/mg protein)

Group A	Group B	p-adj (Tukey)	Significance
Intact	AIA-CFA	<0.0001	***
AIA-CFA	Indomethacin	<0.0001	***
AIA-CFA	Etoricoxib	<0.0001	***
AIA-CFA	Betamethasone	<0.0001	***
AIA-CFA	Combo-L	<0.0001	***
AIA-CFA	Combo-H	<0.0001	***
Etoricoxib	Betamethasone	<0.0001	***
Etoricoxib	Combo-L	0.0047	**
Etoricoxib	Combo-H	<0.0001	***
Betamethasone	Combo-L	0.7501	ns
Betamethasone	Combo-H	0.7931	ns

S2B. IL-1β (pg/mg protein)

Group A	Group B	p-adj (Tukey)	Significance
AIA-CFA	Intact	<0.0001	***
AIA-CFA	Indomethacin	<0.0001	***
AIA-CFA	Etoricoxib	<0.0001	***
AIA-CFA	Betamethasone	<0.0001	***
AIA-CFA	Combo-L	<0.0001	***
AIA-CFA	Combo-H	<0.0001	***
Etoricoxib	Betamethasone	0.0068	**

Group A	Group B	p-adj (Tukey)	Significance
Etoricoxib	Combo-L	0.0891	ns
Etoricoxib	Combo-H	0.0026	**
Betamethasone	Combo-L	0.9651	ns
Betamethasone	Combo-H	0.9999	ns

S2C. IL-6 (pg/mg protein)

Group A	Group B	p-adj (Tukey)	Significance
AIA-CFA	Intact	<0.0001	***
AIA-CFA	Indomethacin	<0.0001	***
AIA-CFA	Etoricoxib	<0.0001	***
AIA-CFA	Betamethasone	<0.0001	***
AIA-CFA	Combo-L	<0.0001	***
AIA-CFA	Combo-H	<0.0001	***
Etoricoxib	Combo-L	0.0566	ns
Etoricoxib	Combo-H	0.0003	***
Betamethasone	Combo-L	0.0003	***
Betamethasone	Combo-H	<0.0001	***

S2D. IL-10 (pg/mg protein)

Group A	Group B	p-adj (Tukey)	Significance
AIA-CFA	Intact	<0.0001	***
AIA-CFA	Etoricoxib	0.0101	*
AIA-CFA	Betamethasone	<0.0001	***
AIA-CFA	Combo-L	<0.0001	***
AIA-CFA	Combo-H	<0.0001	***
Intact	Combo-H	0.8156	ns
Etoricoxib	Combo-H	<0.0001	***
Combo-L	Combo-H	0.0364	*

S2E. COX-2 (ng/mg protein)

Group A	Group B	p-adj (Tukey)	Significance
AIA-CFA	Intact	<0.0001	***
AIA-CFA	Indomethacin	<0.0001	***

Group A	Group B	p-adj (Tukey)	Significance
AIA-CFA	Etoricoxib	<0.0001	***
AIA-CFA	Betamethasone	<0.0001	***
AIA-CFA	Combo-L	<0.0001	***
AIA-CFA	Combo-H	<0.0001	***
Etoricoxib	Betamethasone	<0.0001	***
Etoricoxib	Combo-L	<0.0001	***
Etoricoxib	Combo-H	<0.0001	***

S2F. PGE2 (ng/mg protein)

Group A	Group B	p-adj (Tukey)	Significance
AIA-CFA	Intact	<0.0001	***
AIA-CFA	Indomethacin	<0.0001	***
AIA-CFA	Etoricoxib	<0.0001	***
AIA-CFA	Betamethasone	<0.0001	***
AIA-CFA	Combo-L	<0.0001	***
AIA-CFA	Combo-H	<0.0001	***
Etoricoxib	Combo-L	0.0220	*
Etoricoxib	Combo-H	0.0001	***
Betamethasone	Combo-L	<0.0001	***
Betamethasone	Combo-H	<0.0001	***

* $p < 0.05$; ** $p < 0.01$; *** $p < 0.001$; ns = not significant. All comparisons by one-way ANOVA followed by Tukey HSD post-hoc test. n = 10/group. Only the most informative pairwise contrasts are shown; full matrix available from the corresponding author on request.

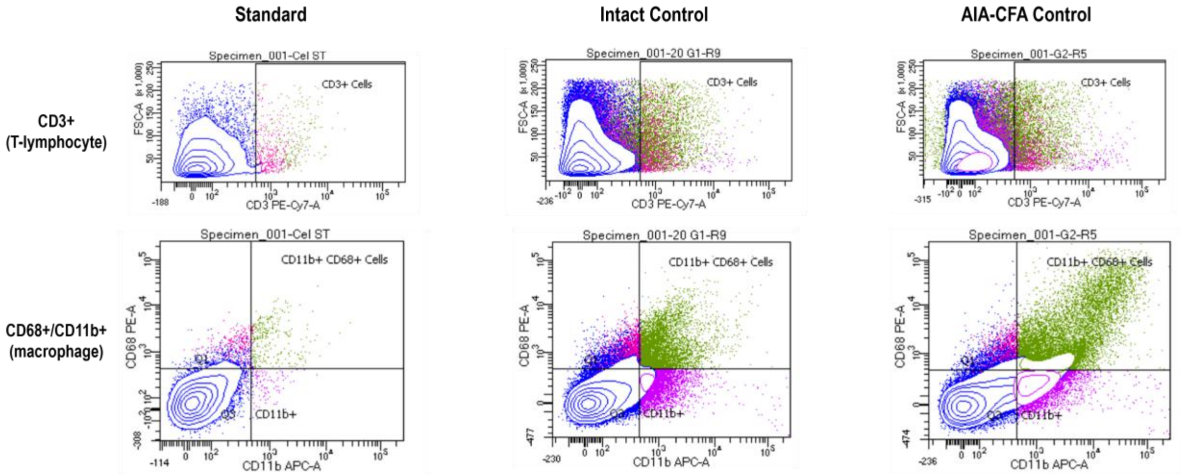

Standard

Experiment Name: SIL-AS-25-002 | Record Date: Nov 29, 2025 9:59:52 PM
Specimen Name: Specimen_001 | SAMPLE ID:
Tube Name: Cel ST

Population	#Events	%Parent	CD68 PE-A Mean	CD11b APC-A Mean	CD3 PE-Cy7-A Mean
All Events	12,644	####	1,054	423	670
All cells	10,399	82.2	336	141	211
Singlets	9,505	91.4	276	117	172
CD3+ Cells	578	6.1	2,119	827	1,709
CD11b+ CD68+ Cells	287	3.0	2,895	1,413	2,287

Intact Control

Experiment Name: SIL-AS-25-002 | Record Date: Nov 29, 2025 8:47:58 PM
Specimen Name: Specimen_001 | SAMPLE ID:
Tube Name: 20 G1-R9

Population	#Events	%Parent	CD68 PE-A Mean	CD11b APC-A Mean	CD3 PE-Cy7-A Mean
All Events	116,238	####	903	1,611	455
All cells	95,278	82.0	303	742	175
Singlets	89,585	94.0	250	539	143
CD3+ Cells	4,412	4.9	2,359	6,455	1,891
CD11b+ CD68+ Cells	5,474	6.1	2,221	1,989	1,136

AIA-CFA Control

Experiment Name: SIL-AS-25-002 | Record Date: Nov 29, 2025 9:08:38 PM
Specimen Name: Specimen_001 | SAMPLE ID:
Tube Name: G2-R5

Population	#Events	%Parent	CD68 PE-A Mean	CD11b APC-A Mean	CD3 PE-Cy7-A Mean
All Events	111,508	####	2,353	3,265	367
All cells	95,406	85.6	1,722	2,302	195
Singlets	90,565	94.9	1,067	1,644	165
CD3+ Cells	4,325	4.8	2,363	12,644	2,148
CD11b+ CD68+ Cells	11,983	13.2	7,240	6,790	573

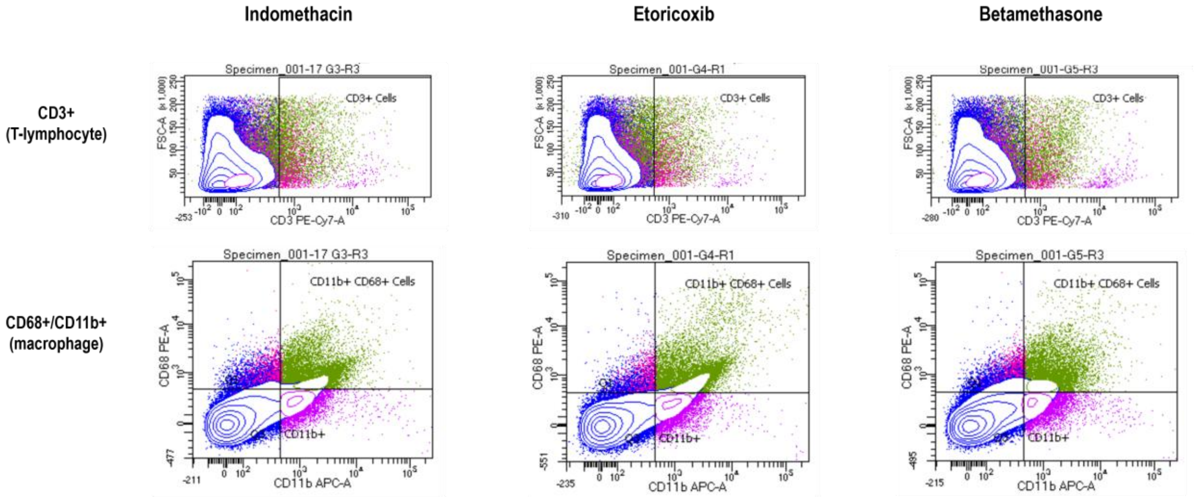

Indomethacin

Experiment Name: SIL-AS-25-002 | Record Date: Nov 29, 2025 8:43:28 PM
Specimen Name: Specimen_001 | SAMPLE ID:
Tube Name: 17 G3-R3

Population	#Events	%Parent	CD68 PE-A Mean	CD11b APC-A Mean	CD3 PE-Cy7-A Mean
All Events	114,995	####	996	1,950	583
All cells	96,447	83.9	363	1,094	232
Singlets	90,381	93.7	279	895	185
CD3+ Cells	4,826	5.3	2,190	10,154	2,291
CD11b+ CD68+ Cells	8,490	9.4	1,685	2,560	1,029

Etoricoxib

Experiment Name: SIL-AS-25-002 | Record Date: Nov 29, 2025 8:59:06 PM
Specimen Name: Specimen_001 | SAMPLE ID:
Tube Name: G4-R1

Population	#Events	%Parent	CD68 PE-A Mean	CD11b APC-A Mean	CD3 PE-Cy7-A Mean
All Events	111,739	####	1,110	1,841	327
All cells	95,827	85.8	637	1,144	153
Singlets	91,109	95.1	416	917	123
CD3+ Cells	3,312	3.6	1,956	11,054	1,986
CD11b+ CD68+ Cells	9,620	10.6	3,007	3,596	566

Betamethasone

Experiment Name: SIL-AS-25-002 | Record Date: Nov 29, 2025 9:03:22 PM
Specimen Name: Specimen_001 | SAMPLE ID:
Tube Name: G5-R3

Population	#Events	%Parent	CD68 PE-A Mean	CD11b APC-A Mean	CD3 PE-Cy7-A Mean
All Events	116,805	####	1,206	2,258	526
All cells	97,786	83.7	534	1,420	242
Singlets	85,159	87.1	372	1,243	192
CD3+ Cells	4,218	5.0	2,188	18,254	2,649
CD11b+ CD68+ Cells	7,983	9.4	2,250	2,158	842

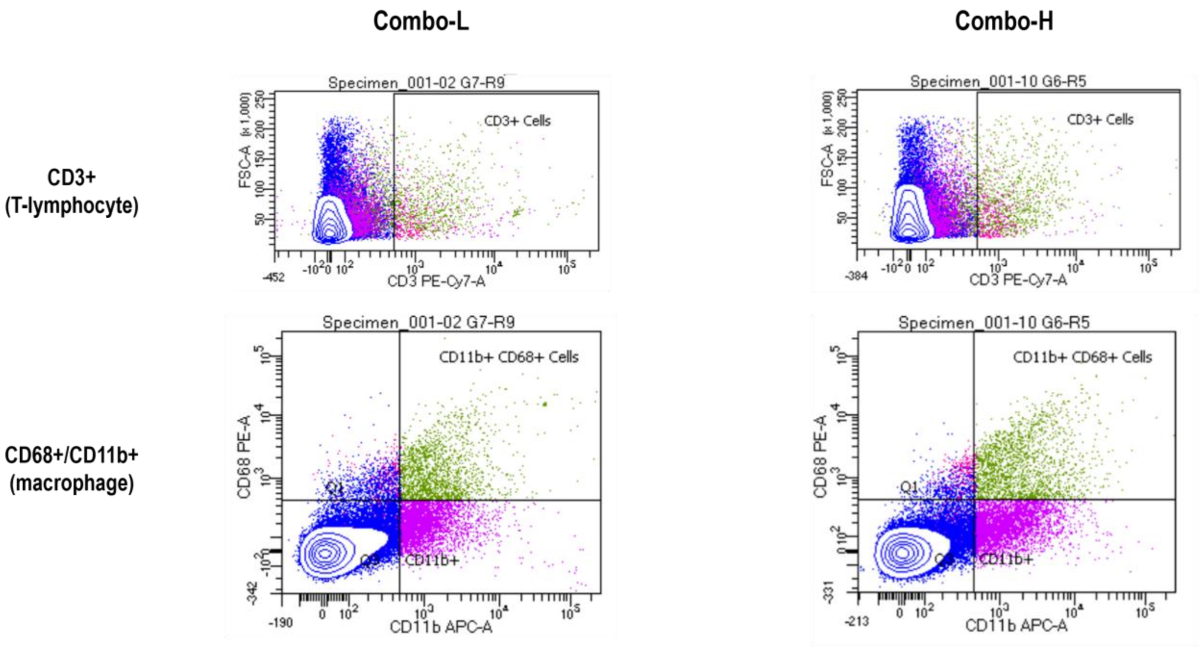

Experiment Name: SIL-AS-25-002 | Record Date: Nov 29, 2025 6:23:44 PM
Specimen Name: Specimen_001 | SAMPLE ID:
Tube Name: 02 G7-R9

Population	#Events	%Parent	CD68 PE-A Mean	CD11b APC-A Mean	CD3 PE-Cy7-A Mean
All Events	106,992	####	364	1,481	250
All cells	90,279	84.4	132	700	94
Singlets	85,926	95.2	103	491	75
CD3+ Cells	1,185	1.4	2,783	20,933	4,035
CD11b+ CD68+ Cells	1,857	2.2	2,681	5,069	2,176

Experiment Name: SIL-AS-25-002 | Record Date: Nov 29, 2025 6:17:56 PM
Specimen Name: Specimen_001 | SAMPLE ID:
Tube Name: 10 G6-R5

Population	#Events	%Parent	CD68 PE-A Mean	CD11b APC-A Mean	CD3 PE-Cy7-A Mean
All Events	104,596	####	312	596	186
All cells	93,924	89.8	121	351	72
Singlets	92,058	98.0	93	300	56
CD3+ Cells	1,402	1.5	2,475	8,742	2,649
CD11b+ CD68+ Cells	1,983	2.2	2,860	4,609	1,531

Figure S1. Representative flow cytometry gating strategy and dot plots for T-lymphocyte (CD3+) and macrophage (CD68+/CD11b+) populations in joint-associated tissue homogenates at study completion (Day 28), presented in three panels: (a) a compensation control sample (“Standard”, used for instrument calibration and fluorescence compensation setup, not a biological/experimental group) together with the Intact and AIA-CFA disease control groups; (b) Indomethacin (5 mg/kg), Etoricoxib (8 mg/kg), and Betamethasone (0.022 mg/kg) monotherapy groups; and (c) Combo-L (etoricoxib 4 mg/kg + betamethasone 0.011 mg/kg) and Combo-H (etoricoxib 8 mg/kg + betamethasone 0.022 mg/kg) combination groups. For each group, the upper dot plot shows the CD3+ T-lymphocyte gate (FSC-A vs CD3 PE-Cy7-A) and the lower dot plot shows the CD68+/CD11b+ macrophage gate (CD68 PE-A vs. CD11b APC-A), with the double-positive quadrant macrophage gate (CD68 PE-A vs (olive green) corresponding to the macrophage population reported in Table 2 and Section 2.6. One representative animal is shown per experimental group (n = 6/group for the flow cytometry sub-study). Accompanying tables report absolute event counts, percentage of parent population, and mean fluorescence intensity (CD68 PE-A, CD11b APC-A, CD3 PE-Cy7-A) for each gated population. Because these panels illustrate the gating strategy in individual representative animals rather than group aggregates, the population percentages shown may differ numerically from the group-level means (mean ± SD, n = 6/group) reported in Table 2.
